# Extreme Evolutionary Conservation of Functionally Important Regions in H1N1 Influenza Proteome

**DOI:** 10.1371/journal.pone.0081027

**Published:** 2013-11-25

**Authors:** Samantha Warren, Xiu-Feng Wan, Gavin Conant, Dmitry Korkin

**Affiliations:** 1 Department of Computer Science, University of Missouri, Columbia, Missouri, United States of America; 2 Department of Basic Sciences, Mississippi State University, Mississippi State, Mississippi, United States of America; 3 Division of Animal Sciences, University of Missouri, Columbia, Missouri, United States of America; 4 Informatics Institute, University of Missouri, Columbia, Missouri, United States of America; 5 Bond Life Science Center, University of Missouri, Columbia, Missouri, United States of America; Duke-NUS Gradute Medical School, Singapore

## Abstract

The H1N1 subtype of influenza A virus has caused two of the four documented pandemics and is responsible for seasonal epidemic outbreaks, presenting a continuous threat to public health. Co-circulating antigenically divergent influenza strains significantly complicates vaccine development and use. Here, by combining evolutionary, structural, functional, and population information about the H1N1 proteome, we seek to answer two questions: (1) do residues on the protein surfaces evolve faster than the protein core residues consistently across all proteins that constitute the influenza proteome? and (2) in spite of the rapid evolution of surface residues in influenza proteins, are there any protein regions on the protein surface that do not evolve? To answer these questions, we first built phylogenetically-aware models of the patterns of surface and interior substitutions. Employing these models, we found a single coherent pattern of faster evolution on the protein surfaces that characterizes all influenza proteins. The pattern is consistent with the events of inter-species reassortment, the worldwide introduction of the flu vaccine in the early 80’s, as well as the differences caused by the geographic origins of the virus. Next, we developed an automated computational pipeline to comprehensively detect regions of the protein surface residues that were 100% conserved over multiple years and in multiple host species. We identified conserved regions on the surface of 10 influenza proteins spread across all avian, swine, and human strains; with the exception of a small group of isolated strains that affected the conservation of three proteins. Surprisingly, these regions were also unaffected by genetic variation in the pandemic 2009 H1N1 viral population data obtained from deep sequencing experiments. Finally, the conserved regions were intrinsically related to the intra-viral macromolecular interaction interfaces. Our study may provide further insights towards the identification of novel protein targets for influenza antivirals.

## Introduction

Influenza type A, a member of the *Orthomyxoviridae* family, is responsible for a large majority of human flu-related illnesses [17], including seasonal epidemics and four documented pandemic outbreaks. The genome is comprised of eight negative-strand RNA segments, encoding 11-12 protein products [18,19]. As a segmented RNA virus, influenza A has two major evolutionary events that define its genomic diversity: replication errors and reassortment [17,20]; these facilitate the emergence of highly pathogenic strains [21–25]. Reassortment, or the exchange of one or more discrete RNA segments into multipartite viruses, occurs frequently between influenza A viruses [26–30] and is critical for the generation of epidemic and pandemic influenza strains. The 2009 H1N1 pandemic virus is a reassortment of genomic segments from distinct swine influenza virus lineages and from human and avian influenza viruses [32]. 

In addition to rapid mutations and frequent reassortments, co-circulating antigenically divergent H1N1 influenza strains significantly complicate vaccine development and use. All these H1N1 viruses have been found to be genetically linked to the 1918 H1N1 pandemic virus [19,31,33,34]. While the hemagglutinin (HA) proteins of the H1N1 virus strains circulating in human populations have evolved considerably since the 1918 pandemic, those in swine have mutated much more slowly [32]. This disparity is evidenced by the structural and antigenic similarities between HA proteins from the 2009 and 1918 outbreaks [19,31,33]. 

The dynamic patterns of the evolutionary changes in the influenza genome are not uniform [17]. One of the best-studied influenza proteins is HA, which has strikingly different patterns of substitution across its sequence, with a handful of residues having high substitution rates [35]. Conservation patterns and their origins in the other influenza proteins are less-studied. Here, we suggest that one source of this variation in substitution rates is the different protein structure context of the residues.

The link between conservation in the influenza proteome and the function of the respective proteins has been studied both computationally and experimentally for several decades [36–40]. Several early studies determined a high sequence similarity between the HA proteins of Influenza A and B, including similarity in the known structural features such as the hydrophobic regions of HA [36]. Another study describing the strong similarity of the epitopes located in the head region of the HA proteins across all subtypes of Influenza A and Influenza B viruses inspired the design of the new broadly neutralizing antibodies [40]. Experimental studies of HA proteins from the H1N1 strains in swine obtained between 1986 and 1991 found the proteins to be highly conserved, both antigenically and genetically [38]. Investigation of the evolution of the influenza A nucleoprotein (NP) gene across five host species using a classical, sequence-similarity based, approach found the evidence of adaptive changes in function among host-specific NPs [37]. Recently, a large-scale study evaluating sequences of 11 viral proteins across Influenza A isolates from avian and human hosts over the last 30 years, isolated 55 conserved sequence fragments with conservation ranging from 80% to 100% and linked many of them to HLA class I or class II epitopes [39]. 

Most of the approaches considered above are sequence-based and do not consider the structural information about the proteins. Several recent approaches, however, have demonstrated that introducing the information about the three-dimensional structure of an influenza protein may provide additional insights into their evolution and specifically the conservation of protein’s structural fragments, which may be sequentially noncontiguous. For instance, two studies reported that structural conservation of human influenza A HA epitope was responsible for interaction with sialoglycans; similarly, conserved influenza B HA epitopes were successfully targeted by the monoclonal antibodies [40,41]. However a structure-based evolutionary analysis of the entire influenza proteome is yet to be done.

Despite their rapid evolution, influenza proteins participate extensively in intra- and inter-species interactions. Human antibodies, for example, recognize at least four antigenic sites on the viral HA protein of H1N1 influenza A virus [42]. Other interactions involve viral macromolecules exclusively, such as PB1, which interacts simultaneously with PA and PB2 [16,43]. More generally, these intra-viral interactions fall into three categories: heteromers, homomers, and protein-RNA interactions. Cataloging the complete human-influenza interactome was a significant challenge, and has been completed only recently [44]. The next step is to better understand the biology of these numerous, newly identified interactions, linking them with the evolutionary mechanisms in influenza. 

In this work, we sought to understand the role of protein three-dimensional structure in the evolution of H1N1 genomes. Specifically, we aimed to answer two questions: (1) whether the surface residues of the majority of H1N1 proteins are diverging faster than are the core residues and (2) whether there are regions of surface residues that are completely conserved, in spite of the anticipated rapid divergence of the protein surface. We began by using a phylogenetic analysis to model the dependence of patterns of amino acid substitutions in the proteins on their exposure to the solvent using the 3D models of the H1N1 proteins. Next, we developed a computational pipeline integrating sequence and structure data in order to identify conserved regions of the proteins’ three-dimensional structures. Each region is a structurally connected “patch” of residues that may not necessarily be sequentially consecutive. The pipeline determines surface residues that are 100% conserved in the sequence alignments, clusters them with respect to their structural positioning, and calculates the probability of observing such a region under a random distribution of conserved residues. Finally, we associated the identified regions with known functional sites, and mapped the mutation sites collected from the viral population data of the pandemic 2009 H1N1 influenza, obtained from deep sequencing experiments.

## Results

### Data collection

The initial set of H1N1 strains included 1,100 unique genomes, each containing ten sequences (*Methods*). We employed a redundancy filter with a whole-genome sequence identity threshold of 95% which yielded a final set of 75 strains (see *Methods*; Table S1), including 10 avian, 34 human, and 31 swine strains, with all strains dating between 1933 and 2009. The 1918 ‘Spanish flu’ strain was not included in the final set due to several of its proteins having 100% sequence identity with the corresponding proteins in other strains, but was included as a case study. Nevertheless, the conservation of the surface regions between the 1918 and 2009 H1N1 pandemic strains was analyzed in detail (see below). The average sequence identity between the individual proteins in our dataset varied from 88.7% to 96.4%. As expected, there were pairs of strains sharing identical or near-identical proteins, even when other proteins in these strains were less than 95% identical. No strains shared the same proteins with less than 60% protein sequence identity (Table 1).

**Table 1:** Strain conservation across the ten proteins.

	HA	M1	M2	NA	NP	NS1	NS2	PA	PB1	PB2
Average	89	97	89	89	95	87	93	96	96	96
Minimum	77	92	71	77	86	60	76	88	86	90
Maximum	100	100	100	100	100	100	100	100	100	100

The proteins vary in their conservation. When removing redundancy we first calculated the pairwise conservation percentage for each individual protein. From this we calculated the average pairwise conservation. We also determined the minimum and maximum conservation.

### Phylogenetic Analysis of Structure-Based Evolution

**Figure 1:**
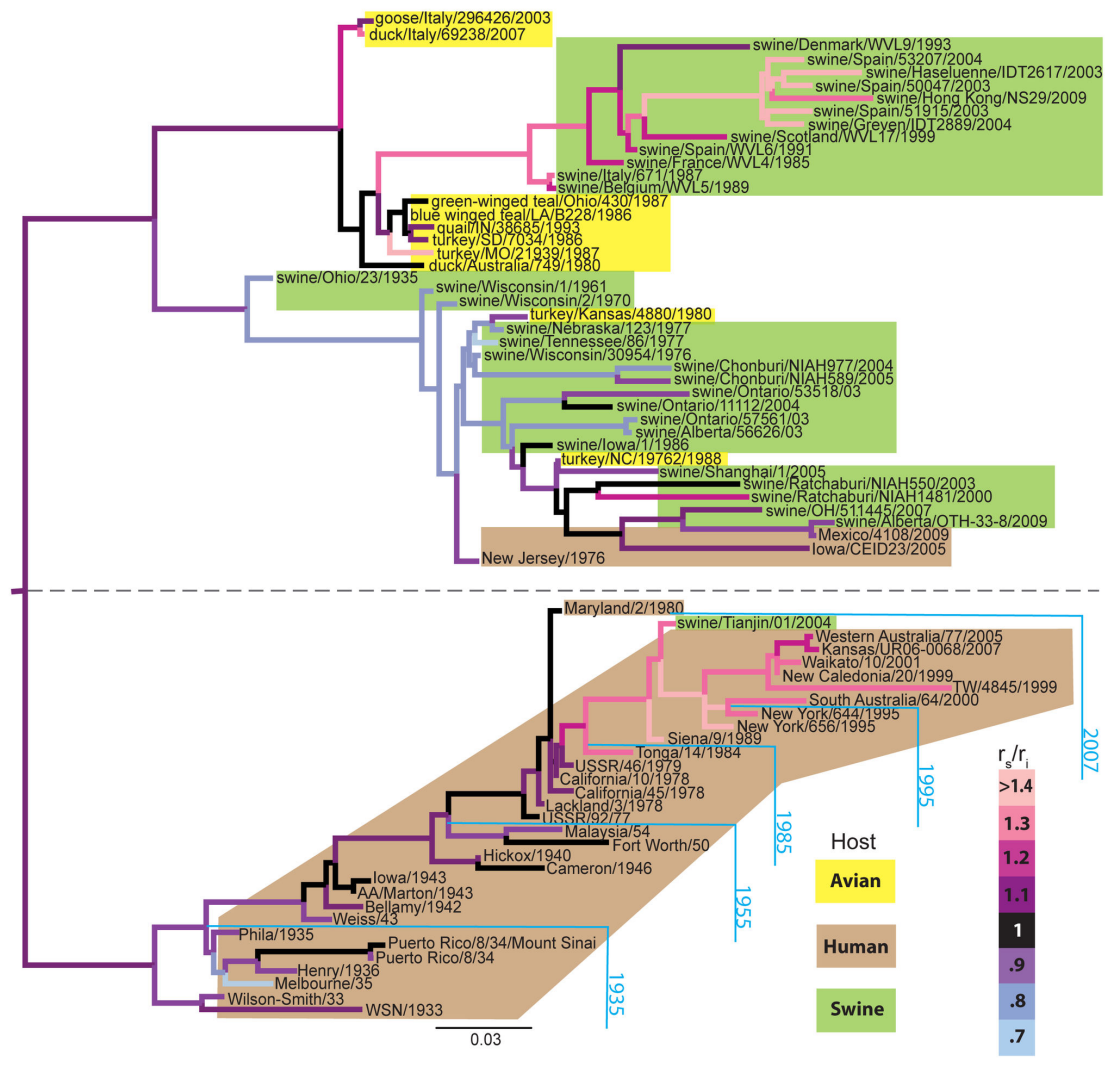
Phylogenetic relationships, species derivation and relative evolutionary rates for 75 accessions of H1N1 influenza. Shown is the topology inferred for the HA protein (see subsection *Inference* *of* *patterns* *of* *molecular* *evolution* *for* *surface* and *interior* *residues* in *Methods*); other proteins show somewhat differing relationships (Supporting Information). We also show the ratio of surface-to-interior amino acid substitutions (r_e_/r_i_), calculated as the difference between the branch lengths estimated from the exterior and interior residues. Variation in r_e_/r_i_ is illustrated from low to high with colors from blue to pink. Each colored box represents the organism of origin: Avian (yellow), Human (beige), and Swine (green). We note that the lower clade (separated by a dashed line) is composed almost entirely of human-derived strains, with the exception of one swine accession (Tianjin/01/2004). This clade also shows a fairly clear timeline (cyan). The upper clade, however, does not give such clear indications of timing.

For each of the ten proteins, we computed the maximum likelihood phylogeny using PhyML [45]. We then fitted several models of evolution to these alignments (*Methods*). The most basic, M_uniform_, requires all nucleotides in the sequence to evolve at the same rate. We compared that model to M_scaled_, where the evolution rate of positions corresponding to surface residues was allowed to be more dissimilar than for interior residues. As expected, all ten proteins showed higher rates of substitution for surface positions (the rate ratio, r_e_/r_i_ was greater than 1.0; Table 2, likelihood ratio test, see *Methods*). We then investigated whether this pattern was the result of differing surface to interior constraints on the various branches of Figure 1, but found no such pattern. Similarly, r_e_/r_i_ varied only slightly for seven of the proteins: 1.1 for NS2; 1.2 for HA, NP, PB1, and PB2; 1.5 for NA and NS1. The ratio was considerable higher for the other three proteins: 1.9 for PA, 2.2 for M1, and 2.3 for M2.

**Table 2:** Protein evolutionary rates and patch information.

Protein	r_e_/r_i_	Exterior / Interior	N of patches	Template coverage	Intra-viral interactions	
					Literature	Structure
HA	1.2	0.53	3	88%	[1]	
M1	2.2	0.63	1	63%	[2,3]	
M2	2.3	6.60	1	38%	[4,5,6,7]	
NA	1.5	0.33	1	82%		3B7E
NP	1.2	0.56	2	94%	[8,9,10]	
NS1	1.5	0.97	1	83%	[11]	
NS2	1.1	1.45	(1)	40%	[12,13,14]	
PA	1.9	0.65	5	91%	[15,16]	2ZNL
PB1	1.2	1.15	(3)	7%		3A1G, 2ZNL
PB2	1.2	0.70	2 (2)	47%	[31]	3A1G

The ratio of protein evolutionary rates for the exterior and interior residues (r_e_/r_i_) was determined using HyPhy. Shown are the ratios for entire proteins. The significant regions are shown in the following column with regions that are biologically significant, but must be explained structurally rather than statistically in parentheses. For some viral proteins the homology models of do not cover the entire sequence due to the limited coverage of their templates. Shown is the percentage of the protein sequence coverage for each structural model. The last column summarizes the evidence for the intra-viral interactions in recent literature and from DOMMINO.

The obtained phylogenetic trees were clearly separated into the host-specific lineages with occurrences of a few strains from other species (Figures 1, S1, S2). The human lineage in both HA and NA trees exhibits a strong ‘trunk-like’ temporal pattern that has been previously observed in the phylogenetic trees generated from whole-sequence alignments [46,47] (Figure 1, S1). In the case of PA, this pattern is less evident (Figure S2). A few human strains were found as a part of the swine clade, and a recent swine strain was found as a part of the human clade across all three analyzed proteins, indicating the bi-dimensional transmission of influenza A viruses between the animal and human interface. Interestingly, we found that after 1984, the surface-to-core ratio of human HA and NA proteins, but not PA proteins becomes significantly higher. This indicates the increasing selective pressure on the surface residues of the former two proteins.

Unlike the human lineage, the swine and avian lineages of HA and NA trees did not exhibit the trunk-like pattern. Instead, the swine lineage was divided into two clades, one comprised primarily of North American strains and another comprised of Eurasian strains. Moreover, while Eurasian swine strains had a surface-to-core ratio that was generally higher than in North American strains, we did not observe the same sudden increase in the ratio values as a function of time, as we did in the human lineages. Finally, several human strains, namely Mexico/2009, Iowa/2005, and New Jersey/1976 were included in swine lineages of HA and NA proteins (Figures 1, S1), representing spillover cases of H1N1 virus from swine to human. This was not necessarily the case for other influenza proteins, which may have originated in different hosts (Figures S2-S9, S11-S13).

### Homology modeling of the individual influenza proteins

The structural analysis of H1N1 protein surfaces using homology modeling is challenged by the limited structural template coverage of some influenza proteins. Three-dimensional structures of several influenza A proteins have been modeled before and used for functional and evolutionary studies [48–52]. Unfortunately, for some influenza proteins (M2, NS2, PB1, PB2) the templates cover only a small portion of the target sequence, while for other influenza proteins the entire sequence is covered by a single template or a number of templates with a little or no structural overlap (HA, M1, NA, NP, NS1, PA). Therefore, we used a single template as the basis for our models for seven proteins and a multiple-template strategy for the remaining three (Table 1). As a result, we obtained models covering almost entire sequences of 6 H1N1 proteins, with the exception of small N-terminal and C-terminal regions. Sequences of 3 proteins were partially covered by two or more fragments (PA, PB1, and PB2). Only one protein (M2) did not have a significant portion of its sequence (residues 23-60) covered by any structural template (Table 1); these regions were not modeled structurally. The average target-template sequence identity was 91% (minimal sequence identity was 45%). This high sequence identity, thus, allowed for an accurate determination of surface and core residues of H1N1 proteins based on the homology models. 

### Conserved regions on H1N1 proteins surface are associated exclusively with intra-viral interactions

**Figure 2:**
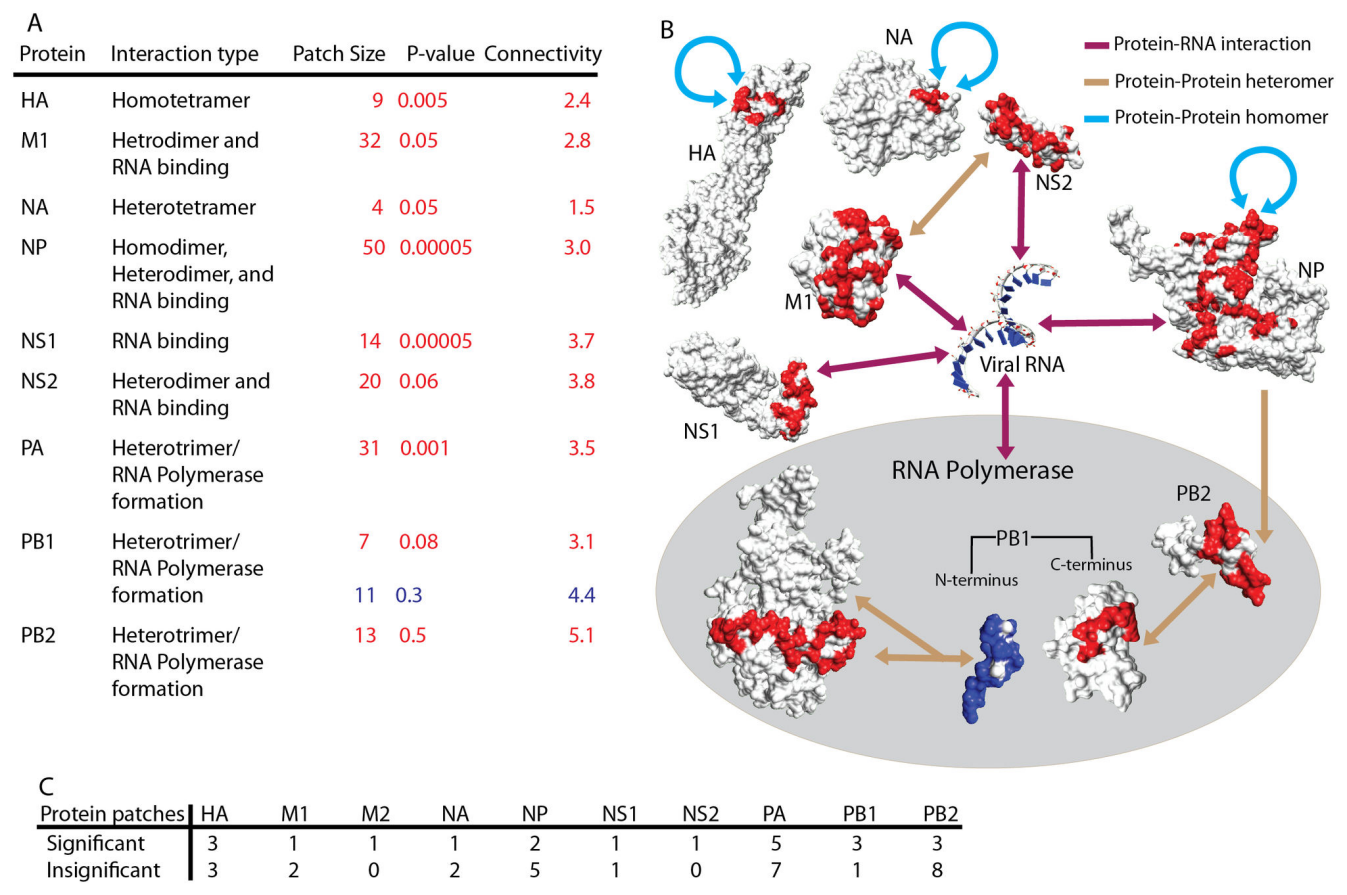
Conserved regions are exclusively associated with known intra-viral interaction positions. A) Eight of the ten viral proteins have regions that are involved in known intra-viral interactions. For each interaction, we list the type of interaction, the size of the patch, E_n_(x), and the patch connectivity. We determine E_n_(x) as the expected number of randomly generated regions of a given size. We calculate the connectivity of the regions as the average number of neighbors each residue has in the patch. The color of the three right-most columns match to the color of the regions in panel B. B) Each of the eight proteins forms a unique interaction with (i) a copy of itself (indicated by a blue arrow), (ii) viral RNA (purple arrow), or (iii) another viral protein (tan arrow). Some conserved regions participate in more than one interaction. A uni-directional arrow indicates an interaction occurring between two proteins, but is not necessarily characterized by conserved regions on both proteins. The three proteins of RNA polymerase, PA, PB1, and PB2, are grouped by a grey oval. Shown is the interaction between the polymerase complex and the viral RNAs. C) The distribution of significant (E_n_(x)≤0.05), marginal (0.05<E_n_(x)≤0.1), and insignificant (E_n_(x)>0.1) regions across all ten proteins.

Each H1N1 protein was found to have at least one evolutionary conserved region that was also statistically significant (Figures 2A, 2C, Table S2). The literature search and a search of DOMMINO database of macromolecular interactions [53] resulted in 8 proteins with regions that had been previously functionally described in the literature (17 papers in total) and 4 proteins that contained regions characterized by structural data (5 PDB structures in total) (Table 2). Even though each protein contained a significant region, some proteins had regions that required structural explanation, such as NS2, PB1, and PB2. The distributions of random patch sizes obtained for these proteins did not fit well using an exponential distribution. Specifically, the distribution of random patch sizes for NS2 closely resembled a linear stepwise function, and for structurally modeled fragments of PB1 and PB2 the underlying distributions favored the regions of maximum size. This can be explained by the large percentage of surface residues that are classified as conserved. Indeed, since a large number of surface residues are conserved, it is difficult to create several isolated regions of small size; thus, the typical regions are large. For M1, we also obtained a random patch size distribution, which appeared almost exponential with the exception of an additional peak. Finally, the M2 protein, with a similarly high substitution rate, had a significant region on its surface. However, the small size of the M2 structural model covers only part of its sequence, possibly giving rise to a spurious patch. The location of the modeled structure in the transmembrane region increases the likelihood of existence of such a patch.

Intriguingly, the functional annotations of the significant regions reveal that all the regions are exclusively associated with the intra-viral protein-protein and protein-RNA interactions (Figure 2B), with the exception of a single residue from a region on NP (Table S2). The protein-protein interactions include both homomers (self-interactions of proteins M1 [3], M2 [5,6], NP [10], and NS1 [11]) and heteromers (interactions mediated by proteins M1 [2], NP [8], NS2 [12,13], PA [15,16], PB1 (PDB: 2ZNL, 3A1G), and PB2 (PDB: 3A1G)). Several of these proteins, including M1 [2], NP [9], NS1 [11], NS2 [14], PB2[31], also had significant surface regions associated with protein-RNA interactions. The conserved regions share several interesting properties. First, we found that all interactions involved in the assembly of the RNA-polymerase complex included at least one region of extreme conservation. Second, while regions usually occurred on only one binding site of the interaction interface, we also found protein-protein interactions with the regions included in both binding sites (interactions between proteins PB1 and PA [16] (PDB: 2ZNL), PB2 and PB1 (PDB: 3A1G), and M1 and NS2 [2,12,13]). Finally, we found that NS2 had a conserved region annotated with multiple functions: the region from residues 65-72 is involved in both viral RNA and M1 binding, while residues 74-79 are involved exclusively in M1 binding (Table 2).

The inferred regions were only slightly affected when we additional sequences were introduced to the original non-redundant set of 75. Specifically, it took between 900 and 1000 sequences to introduce even a single non-synonymous polymorphism into a single influenza protein. Most strains experienced exactly one such mutation across their entire proteome. A particular set of outlying strains caused at most 5 polymorphisms and affected at most 3 different proteins (Table S3). This set contains 14 proteomes that can be grouped by geographic location and close years, and within these groups, the sequence identity ranges from 97% to 100%. This indicates that these grouped sequences are in the same redundancy cluster, during the redundancy removal procedure and thus could have only a minimal effect, if any, on the analysis.

### Regions of extreme conservation in 1918 and 2009 pandemics

Following the findings by Xu et al [33], which identified nearly identical functional sites shared between HA proteins of the 1918 and 2009 H1N1 pandemics, we compared our identified regions of extreme conservation across strains from both pandemics. Notably, all identified regions across all proteins were identical between the 1918 and 2009 strains. This finding is in agreement with the fact that the 2009 swine origin pandemic influenza A virus is thought to originate from a recent inter-species reassortment from swine to human, and another observation that same extreme regions were found not only between human H1N1 strains but also across swine and avian strains.

**Figure 3:**
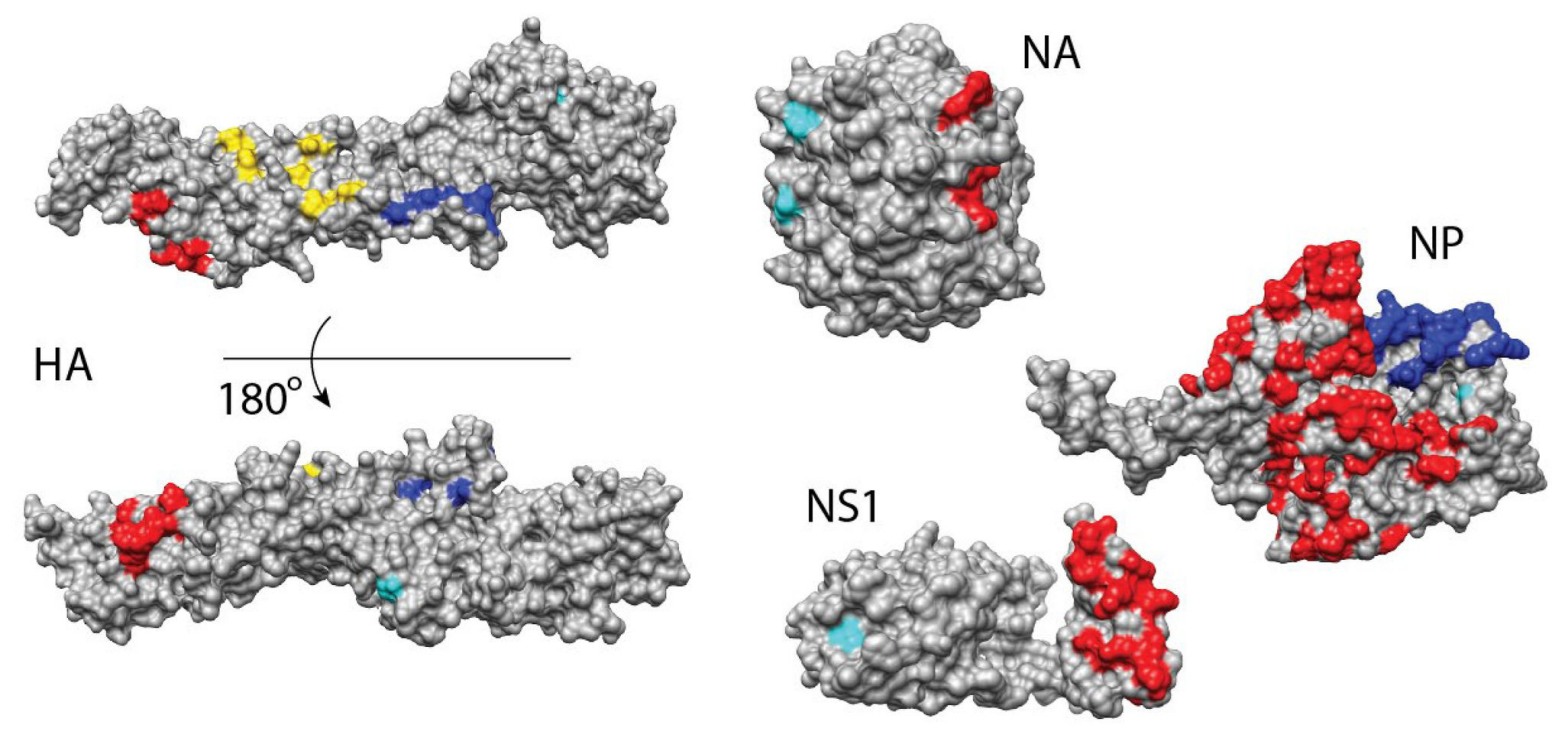
Genetic variation of the viral population data obtained from a patient does not affect regions of extreme conservation. Shown are ten mutation sites (cyan) from four proteins, HA, NA, NS1, and NP, obtained using a deep sequencing approach. The mutation sites were mapped onto the structural models and their locations were compared to the conserved regions. Individual regions of extreme conservation were coloured red, blue and yellow.

We finally sought to understand the relationship between the identified regions of extreme conservation and the evolutionary dynamics of the virus when treated with antiviral drugs. Specifically, we used recently reported viral population data obtained from an immunosuppressed patient infected with 3 variants of H1N1/2009 influenza and treated with neuraminidase inhibitors [54]. The data included a set of ten mutation sites from four proteins obtained using a deep sequencing approach: HA (Val_6_, Asn_55_, Val_125_, Thr_220_), NA (Ile_106_, Asp_199_, Asp_248_, His_275_), NP (Ile_100_), and NS1 (Ile_123_). These sites were mapped onto the homology models of the proteins and compared to locations of conserved regions (Figure 3). We found that none of the ten mutation sites belonged to any of the conserved regions. Interestingly, NA’s mutation site Ile_106_ was in close proximity to residues 107 and 108, which belonged to a conserved region. However, the mutation reported at this position (I106V) [54] is unlikely to cause any changes in the function associated with the conserved region due to similar properties of the residues.

## Discussion

### Overview of the addressed problem and result highlights

**Figure 4:**
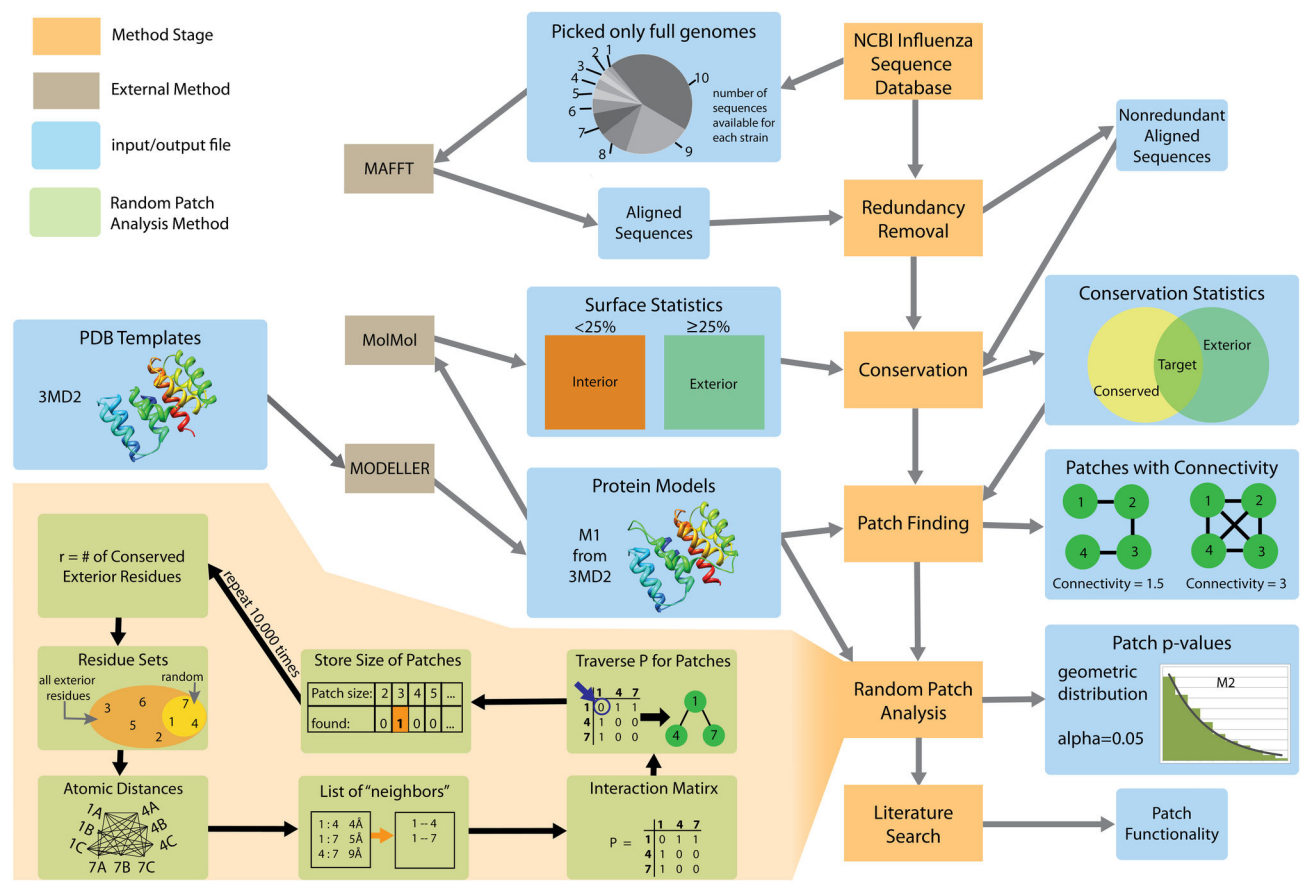
Method pipeline. Our conserved patch analysis method consists of six stages (orange boxes): data collection, redundancy removal, conservation detection, patch finding, random patch analysis, and functional annotation of the conserved regions. The method integrates data from multiple sources (blue) and employs four previously developed software packages (grey): MAFFT, MolMol, and MODELLER. The random patch analysis stage is described in more detail (peach).

The conservation of functionally important residues on protein surfaces has been well documented [55,56]. In particular, several studies, both general and targetting specific protein families, determined the sequence and structure conservation of residues in the protein binding sites mediating intra-species protein-protein interactions [55,57,58]. However, the impact of the purifying selection on the protein binding sites in viral proteins is not clear, due to the intrinsic relationship between the intra-viral and viral-host protein-protein interactions. Unexpectedly, we gained new insight into the evolution of viral binding sites while addressing more general questions related to influenza protein evolution. The first question is whether the surface residues of the proteins evolve faster than the core residues, and whether this is seen equally across all influenza proteins. The second question is whether, in spite of the rapid evolution of surface residues in influenza proteins, there are any “extreme” protein regions that are fully conserved. To answer these questions, our approach integrated the data from evolutionary genomics, structural bioinformatics, and deep sequencing. The developed automatic pipeline (Figure 4) has allowed for the first time to detect statistically significantly conserved regions in the entire influenza proteome that are structurally connected but may not necessarily be sequentially contiguous. The pipeline is readily available to study proteomes of other viral families.

### Evolutionary dynamics of H1N1 and our hypothesis

It was recently shown that reassortment with swine strains resulted in nearly identical regions of conserved antigenic residues in HA protein of the 1918 and 2009 H1N1 strains [33,59]. However, that conservation is in striking contrast to the 50% sequence divergence between strains from 2007 and the 1940's [33] and appears the result of the replacement of H1N1 genes from the human strains with those from swine strains, which are much slower evolving in the protein sequence [32]. This combination of rapid evolution and reassortment is the principal reason for the lack of conserved regions around the HA antigenic sites, when considering H1N1 strains of different years. The result points to a more general conclusion: the evolutionary conserved surface regions, should any exist, are unlikely to occur in the regions mediating the viral-host interactions, for which the host proteins may be subject to selection against viral replication. Indeed, host-viral interactions may give rise to Red-Queen/arms-race type dynamics [60]. 

### Insights to obtained exterior-to-interior evolutionary rates across different proteins

In addition to confirming a higher rate of evolution on the surface of viral proteins when compared to the interior, our phylogenetic study revealed signals of viral reassortment in influenza strains from other hosts {Brown, 2000 #37;Li, 2005 #1580;. As a result, each protein has a unique gene-tree topology (although we did not assess the phylogenetic uncertainty inherent in these trees, since the tree inference was not a primary goal of our study). The source of the variation in exterior-to-interior residue rate ratios (r_e_/r_i_) is less straight-forward to explain. While most values were between 1.1 and 1.5, PA (1.9), M1 (2.2), and M2 (2.3) were significantly higher. One possible reason is that PA and M2 were both incomplete structures, thus residues that are buried in the full structure could be assigned as "exterior" residues. Thus, structural data for M2 was limited to the helix-linker-helix structural fragment of the transmembrane region, resulting in 33 “exterior” residues and only 5 “interior” residues, even though all of these residues would be buried in a membrane *in vivo*. 

### Structure-based phylogenetic analysis provides insights into the multi-species evolution of H1N1 virus

Using structure-driven phylogenetic analysis, we found that the human lineage of HA and NA phylogenetic trees of the H1N1 virus had a trunk-like structure while swine and avian lineages did not, indicating that the topological diversities of phylogenetic trees for H1N1 viral proteins can reflect the difference of selective pressures in human and animals. Indeed, due to a longer life span and fewer limitations on geographical barriers, the human influenza virus can be further exposed to herd immunity. As a result, one strain can be easily circulated globally. On the other hand, multiple sublineages of influenza viruses can be co-circulating in different and geographically separated animal populations. In contrast to the surface proteins, the human lineage of internal H1N1 proteins, e.g. PA, do not have trunk-like structures. This is likely due to the frequent reassortments {Nelson, 2008 #1539;Zinder, 2013 #1540}, and these proteins can have different animal origins and evolutionary histories. 

The fact that there are viruses from multiple hosts located at the same lineage indicates frequent bi-dimensional transmission of influenza A viruses at human-animal, and animal-animal interfaces. For example, Mexico/2009, Iowa/2005, and New Jersey/1976 are three well-documented swine-origin influenza A viruses [61–63]. Nevertheless, the comparative analysis of the structural patterns in the phylogenetic trees of individual proteins suggests that these reassortments were different in their nature: for HA, all three strains are clustered together within North American swine lineage; for NA, Iowa/2005 and New Jersey/1976 strains are clustered with North American, while Mexico/2009 is clustered with European clade; finally for PA, Iowa/2005 and Mexico/2009 are clustered with a larger clade that includes avian and European swine lineages, while New Jersey/1976 is clustered together with other human strains.

An interesting feature of the human lineage is that the surface-to-core ratios of HA and NA proteins have increased significantly since 1984 (Figs.1, S1). Such increase could be due to H1N1-specific herd immunity from accumulating infections of H1N1 since 1977 as well as vaccine-derived immunity, as the first nation-wide vaccination was introduced in the U.S. at the end of 1976 [64,65]. This observation was only among the surface proteins HA and NA, but not internal proteins because HA and NA are the primary target of the immunological system.

Finally, when comparing the surface-to-core rates between Eurasian and North American swine lineages, two differences were noticed. The first difference, the fact that Eurasian swine lineage is clustered together with the avian lineage, while North American swine lineage is not, can be explained by the well-documented multiple transmission events of the avian H1N1 virus to pigs in continental Europe and later in Asia [66–68]. The second difference, the consistently higher surface-to-core ratios in Eurasian swine lineage, compared to the North American lineage, has not been previously reported. One explanation may be that unlike the classical swine flu in North American lineage, the swine influenza virus from the European lineage, once transmitted from the avian host, required fast adaption to the swine host. In addition, the rate difference may be associated with the suggested difference in epizootiology between the U.S. and European swine influenza, since in Europe herds may harbor the virus while showing no clinical symptoms [69,70]. A further analysis with a more detailed reassortment history between the avian and swine lineages may be required to confirm this hypothesis.

We note that sampling bias of the strains could also be an influencing factor in our analysis, since it is one of the most common problems in influenza sequence analysis in general. For instance, the most diverse of large clusters of similar strains defined during the redundancy removal is likely to have more random mutations than those of small clusters. Thus, the higher r_e_/r_i_ ratio of the Eurasian swine lineage compared with the North American lineage could be a byproduct of sampling bias. Unfortunately, sampling bias is difficult to avoid, so one should be cautious not to overinterpret the changes of r_e_/r_i_ ratio over time in such cases. To handle sampling bias, several approaches could be explored in the future. For instance, one could look at the correlation of the r_e_/r_i_ ratios of strains with the number of redundant strains they represent or at the average values of r_e_/r_i_ ratio per year versus number of samples in the dataset before and after redundancy removal for the same year.

### Effects of positive and negative selection on the protein surface in H1N1 proteins

While all of the virus proteins are subject to evolutionary change, the extent to which each protein allows certain changes depends on several factors such as location of the protein in the virion, the protein’s function, and the fact that some genes are encoded on the same genomic segment. For instance, HA is expressed on the surface of the virion, is involved in host binding, and is located on its own gene segment [71]. Thus, HA is subject to a stronger selective pressure compared to the internal proteins, such as M1, which serves a structural purpose as well as RNA binding, and shares a coding region with M2 [71]. Because of this shared coding region, each mutation risks causing a detrimental change in the other gene. There is also variation within a given protein: HA’s antigenic sites are subject to positive selection due to host immune pressure, yet the stem region is subject to purifying selection due to its role in trimer formation. This mixed selection is seen in essentially all of the proteins: there exist regions that are subject to positive selection due to their role in viral-host interactions and there exist regions that are subject to negative selection due to their role in intra-viral interactions.

### High conservation of H1N1 functional regions have been previously reported

There have been several studies that have found high but not necessarily 100% conserved regions on the surfaces of the influenza proteins. For instance, it has been found that the dsRNA binding track of NS1 consists of conserved binding residues [72]. Additionally, the conservation of the surface regions has been determined near the stem region of HA protein, [73]. Since HA evolves considerably faster than NS1, it is of note that both of these structures are known to have conserved binding regions. The regions found in the present study overlapped with regions identified, experimentally, to be conserved but did not overlap with them entirely. 

### Analysis of extremely conserved regions

In concert with the above findings, we found that all of the detected conserved regions were associated with the intra-viral macromolecular complexes, including protein homomers, heteromers, or protein-viral RNA interactions. Interestingly, each region covered a part of, but never the entire, binding site. This type of co-localization suggests that though most of an intra-viral binding site is conserved, variable residues exist perhaps under weaker selective pressure than their conserved neighbors. In the case of M1, NP, and NS2, the conserved regions are co-localized with multiple binding sites. Note that each of these interactions buries the exposed residues of conserved regions in the interaction interface, effectively making them the interior residues. However, while some interactions are more long-term than others, none are bound for the entire viral life cycle. In contrast to the situation with host-viral interactions, natural selection is expected to stabilize intra-viral interactions [74], which accounts for their conservation. Alternatively, there could be co-evolution between the interacting residues, such as found in some host-viral interactions [75,76]. While each significant region has been associated with at least one known functional region, there are portions of each region that do not overlap with any functional sites. Those regions may be involved in undiscovered intra-viral interactions. This hypothesis is plausible, given that very few known interactions have been comprehensively characterized on the residue level. The geographic scale and time scale, together with the degree of observed extreme conservation in the influenza proteins allows one to suspect that these conserved regions would also occur across viral strains in any given year. Consistent with this hypothesis, our mapping of genetic variation obtained from an individual carrying three genetic variants from two distinct phylogenetic clades did not find a single mutation in any of the conserved regions. However, further studies involving multiple subjects and larger viral populations are necessary to provide a stronger linkage between the temporal and population-wise conservation of the functional regions in influenza proteins.

### Our findings may provide insights into new influenza drug targets

Attaining total protection against Influenza A virus through the development of universal antivirals and vaccines has been a challenging task due to the increasing resistance to the treatments of new viral strains as well as the enormous diversity of the viral population. Recently, a number of promising approaches have been identified, including human monoclonal antibodies and antivirals inhibiting the activity of influenza proteins. Both vaccines and antiviral are capable of neutralizing a wide range of influenza A and often B strains [77–83], but they have been focused thus far on only a few protein targets: the vaccines for HA and antivirals for M2 and NA. Moving beyond these targets, the design of new protein inhibitors of influenza polymerase has been recently suggested as a potential direction in the development of new antivirals [84]. Our study may provide further insight towards identifying new protein targets for influenza antivirals or antibodies, pinpointing the key binding regions that are conserved across a wide range of current and past influenza strains and thus likely to be preserved in future strains. One example from our data is the PB1 to PB2 interaction, which, if disrupted, could result in the loss of viral RNA replication function [85]. One of the main challenges in targeting the regions of extreme conservation, however, comes from their intrinsic property: the regions become inaccessible upon intra-viral macromolecular interactions. Understanding the dynamics of such interactions may provide further insight into this challenge as well as the evolutionary mechanisms behind the extreme conservation.

## Methods

### Data selection and alignment

Our data selection protocol was carried out in three stages. First, a set of 1,100 complete genomes of H1N1 influenza was selected from the NIH Influenza Virus Resource (Table S1). All 100% identical sequences were filtered. Because most genomes had only PB1-F2 sequence fragments, we chose to use only the other ten proteins. During the second stage, the redundant strains were identified: we defined two strains as redundant if the sequence identity for each of the ten pairs of proteins was greater than 95%. Sequence identity was calculated based on the sequence alignment obtained using MAFFT [86]. Finally, the strains were clustered into redundancy clusters, relative to their redundancy with each other, and a representative was selected for each cluster, resulting in 75 non-redundant strains (Table S1). Using the remaining 1,025 sequences, we analyzed how the addition of sequences to the non-redundant set of 75 affected site-specific conservation. This analysis was also done using multiple sequence alignment software MAFFT [86]. 

### Protein Structure Prediction and Surface Analysis

The accurate identification of the surface residues for each influenza protein is a critical step in our approach. The ideal method for inferring each surface residue is to compute a homology model for each protein sequence and using the model structure to define the accessible surface residues. However, making such inferences for each sequence is computationally expensive. Therefore, in our protocol, a single target sequence was randomly chosen from the selected strains of each of the ten proteins, and a corresponding protein structure was predicted using the comparative modeling software MODELLER (Table 3) [87]. Next, for each modeled protein, we identified exterior residues using the CalcSurface subroutine. This routine calculates the solvent accessible surface area (SASA) using the MolMol software package [88]. Residues with a SASA greater than 25% were defined as exterior. This threshold has been previously used to identify a protein’s surface residues [89]. Finally, the surface residues of the remaining 74 strains were mapped from the modeled strain using sequence alignment.

**Table 3:** Coverage and sequential similarity of protein templates.

Protein	Strain	Template	Template subtype	Template similarity, %	Residues covered
HA	A/Fort Worth/50	1H0A (A)	-	45	19-517
M1	A/Iowa/1943	1AA7 (A)	-	97	1-158
M2	A/Iowa/1943	2KIH (A)	H5N1	89	23-60
NA	A/Iowa/1943	3B7E (A)	H1N1	91	83-467
NP	A/swine/Alberta/ OTH-33-8/2009	2Q06 (A)	H5N1	93	28-502
NS1	A/Fort Worth/50	3F5T (A)	H5N1	90	5-202
NS2	A/Iowa/1943	1PD3 (A)	H1N1	100	68-116
PA	A/Iowa/1943	3HW3 (A)	H5N1	96	1-193
		2ZNL (A)	H1N1	96	239-699
PB1	A/Iowa/1943	2ZNL (B)	H1N1	100	1-15
		3A1G (A)	H1N1	95	686-736
PB2	A/Iowa/1943	2ZTT (B)	H1N1	94	1-36
		2VQZ (A)	H3N2	95	318-457
		3R2V (A)	H3N2	93	538-720

To make the protein models, we first selected a sequence and one or more templates for each protein (many of the proteins needed multiple structures in order to cover most of the sequence). To select the templates for PA, PB1, and PB2 we chose the PDB references with the highest coverage and best resolution. For the others, we used MODWEB, which will automatically pick the best template. We picked the sequence (or strain) based on the sequence alignment. We generally selected either the sequence with the least number of gaps or the smallest number of unique gaps. The sequence similarity between the template and sequence is significantly high due to the high conservation between strains (Table 1).

### Inference of patterns of molecular evolution for surface and interior residues

Using the structural information obtained from the comparative modeling, we explored the difference between patterns of sequence evolution of the proteins' interior and surface residues. Specifically, we fit three models of sequence evolution to these data using maximum likelihood, as implemented in the HyPhy software package [90]. The first and most restrictive model *M*_*uniform*_ requires that the estimated branch lengths of the surface and interior partitions be identical. Thus, this model allows for no overall difference in the rate of evolution between the surface and interior residues. In the second model, *M*_*scaled*_, we relaxed this assumption slightly to allow two partitions to have branch lengths that differ by a scaling constant α. Thus, each branch length for the surface partition is multiplied by α (generally <1.0) to give a corresponding length for the interior partition. In the third model, *M*_*arbitrary*_, the branch lengths of the two partitions are estimated completely independently. We note that our models do not explicitly take into account rate heterogeneity. Phylogenetic analyses typically treat rate heterogeneity as a poorly understood nuisance parameter [91]. However, as we have previously discussed, a significant contributor to this variation is the variation between surface and interior residue selective constraint [92,93] that we have accounted for with our structural models.

These three models are nested with respect to each other, with model *M*_*uniform*_ being a special case of both model *M*_*scaled*_ (when α=1.0) and *M*_*arbitrary*_ (when the paired branch lengths for the two partitions are equal). We can thus use a likelihood ratio test [94] to ascertain whether *M*_*scaled*_ constitutes a statistically significant improvement over the null model *M*_*uniform*_. The likelihood ratio compares the difference in log-likelihood between the two models to a chi-square distribution, where the number of degrees of freedom of that distribution is given by the number of excess parameters in the alternative model. For *M*_*scaled*_, the parameter α adds one degree of freedom. Therefore, if the above test shows significant improvement for *M*_*scaled*_, one can then explore whether the model may be further improved by allowing each branch to differ between the surface and interior residues (i.e., model *M*_*arbitrary*_). We again used the likelihood ratio test: in this case there are 146 extra parameters, corresponding to the 147 extra branch lengths in *M*_*arbitrary*_, minus the unnecessary α parameter.

### Automated conservation analysis pipeline

We next developed an automated computational pipeline to determine structurally conserved protein regions and assess their statistical significance. This pipeline was applied to study the extremely conserved regions of the H1N1 proteome (Figure 4) and consists of four basic steps. We first determine the conserved residues shared between a set of representative protein sequences. Second, we use the homology models of the H1N1 proteins to filter out the conserved residues in the core of each protein. Third, we cluster the remaining residues that are fully conserved on the surface into regions. Finally, we determine the statistically significant regions by employing a random model that generates surface regions with similar properties. The process is further described below.

To identify the regions of extreme conservation, we aligned the set of 75 representative sequences for each of the 10 proteins and determined which of the surface residues were 100% conserved across all 75 sequences. Next, we calculated the Euclidean distance between all pairs of 100% conserved exterior residues. Pairs of conserved residues that were no farther than 6Å apart were defined as structural neighbors. The neighborhood relationship was then summarized as a binary contact matrix of a graph, and the whole set of surface residues were represented as a neighborhood graph with edges designated by the contact matrix. Finally, the surface residues were clustered into regions by defining each connected component of the neighborhood graph to be a cluster. In addition, for each region we calculated its size, contributing surface residues, and residue connectivity. The residue connectivity is defined as an average number of edges per vertex in the neighborhood graph.

To assess if the sizes of the observed regions were larger than expected by chance, we generated a sample of random patches using the corresponding MODELLER subroutine [95]. For each sample, the procedure randomly selects the same number of unique surface residues as conserved surface residues on the protein structure. We then apply the same clustering algorithm as the one discussed above to each of the randomly generated samples, obtaining a patch of neighboring residues and determining the size of the patch. We repeated this procedure 10,000 times (the number is selected as a trade-off between the sample size and computational time of the random trial procedure), yielding a distribution of patch sizes expected for randomly selected groups of exterior residues. The conserved regions obtained from the real data were compared against this distribution, identifying significant regions. Specifically, we determined the *P*-value for each region size using a geometric distribution with a weighted average: 

$$P-value=1-{\left(1-\frac{1}{avg(X)}\right)}^{n},\;avg(X)=\frac{\sum_{n=1}^{L}n*y_{n}}{\sum_{n=1}^{L}y_{n}},$$

where *X* is the set of all random patches and frequencies, *n* is conserved region size, *y*_*n*_ is the frequency of a patch of size *n*, and *L* is the largest possible patch. For this weighted average, we also considered patches of size 1, the residues that were isolated after clustering. The addition of these residues was necessary for understanding the underlying distribution. The distribution appeared exponential, however since the distribution was of discrete values, we decided that a geometric distribution was a better choice. We then defined a region as significant if the *P*-value was less than 0.05. 

Each statistically significant region was functionally annotated. Specifically, we mapped intra- and inter-species binding sites of the H1N1 influenza proteins collected from our database of macromolecular interactions DOMMINO [53] and PubMed literature search, and then determined if each of the conserved regions overlap with any of the mapped binding sites (see Table 2).

## Supporting Information

Text S1 **Information on conserved regions in influenza proteins.**
(DOCX)

Figure S1 **Phylogenetic relationships, species derivation and relative evolutionary rates inferred for the NA protein based on 75 accessions of H1N1 influenza.** Shown are the inferred topology and the ratio of surface-to-interior amino acid substitutions (r_e_/r_i_), calculated as the difference between the branch lengths estimated from the exterior and interior residues. The coloring scheme is the same as in Figure 1.
(TIF)

Figure S2 **Phylogenetic relationships, species derivation and relative evolutionary rates inferred for the PA protein based on 75 accessions of H1N1 influenza.** Shown are the inferred topology and the ratio of surface-to-interior amino acid substitutions (r_e_/r_i_), calculated as the difference between the branch lengths estimated from the exterior and interior residues. The coloring scheme is the same as in Figure 1.
(TIF)

Figure S3 **Phylogenetic relationships and relative evolutionary rates inferred for M1.** **The** inferred topology and the ratio r_e_/r_i_ are calculated as in Figure 1.
(TIF)

Figure S4 **Phylogenetic relationships and relative evolutionary rates inferred for M2.** The inferred topology and the ratio r_e_/r_i_ are calculated as in Figure 1.
(TIF)

Figure S5 **Phylogenetic relationships and relative evolutionary rates inferred for NP.** The inferred topology and the ratio r_e_/r_i_ are calculated as in Figure 1.
(TIF)

Figure S6 **Phylogenetic relationships and relative evolutionary rates inferred for NS1.** The inferred topology and the ratio r_e_/r_i_ are calculated as in Figure 1.
(TIF)

Figure S7 **Phylogenetic relationships and relative evolutionary rates inferred for NS2.** The inferred topology and the ratio r_e_/r_i_ are calculated as in Figure 1.
(TIF)

Figure S8 **Phylogenetic relationships and relative evolutionary rates inferred for PB1.** The inferred topology and the ratio r_e_/r_i_ are calculated as in Figure 1.
(TIF)

Figure S9 **Phylogenetic relationships and relative evolutionary rates inferred for PB2.** The inferred topology and the ratio r_e_/r_i_ are calculated as in Figure 1.
(TIF)

Figure S10 **Overlap of binding sites and conserved regions of HA and NA proteins.** Shown are conserved regions (blue), protein binding sites (gold) and the overlap of binding sites and conserved regions (red).
(TIF)

Figure S11 **Phylogenetic relationships and relative evolutionary rates inferred for HA, protein shown with bootstrap values.**
(TIF)

Figure S12 **Phylogenetic relationships and relative evolutionary rates inferred for NA protein shown with bootstrap values.**
(TIF)

Figure S13 **Phylogenetic relationships and relative evolutionary rates inferred for M1 protein shown with bootstrap values.**
(TIF)

Table S1 **A representative set of 75 strains used in our analysis.**
(DOCX)

Table S2 **Conserved regions with p-value and residues.** Each protein is shown with their associated regions, p-values, and residues. Residues in bold are also found in intra-viral binding regions.
(DOCX)

Table S3 **Outlying strains and the regions they affect.**
(DOCX)
