# Supplementary material for: Extreme Evolutionary Conservation of Functionally Important Regions in H1N1 Influenza Proteome
Source: PLoS One. 2013 Nov 25;8(11):e81027. doi: 10.1371/journal.pone.0081027 (PMC3839886; doi:10.1371/journal.pone.0081027)
Supplement: Text S1 — Information on conserved regions in influenza proteins. (DOCX) [file pone.0081027.s001.docx]

# SUPPORTING INFORMATION TEXT

## Conserved regions in HA

We found three significant regions on the surface of HA: one of size 9 (p-value = 0.005, connectivity 2.4) and two of size 7 (p-value = 0.02, connectivity 2.8 and 1.7). Each of these regions overlaps with the homotrimer interface on the stem region [1]. Interestingly, these interactions occur between HA2 domains only. Using DOMMINO we found that the region of size 9 consists of residues from one interface (PDB:1EO8) and the two regions of size 7 consist of residues from the other interface (PDB:1HGD).

## Conserved regions in M1

On the surface of the M1 protein we found a region of size 32 (p-value = 0.05, connectivity 2.8). This region is comprised of a linker, a middle section, and the end of the N-terminal of M1 [2]. The region consisting of residues 101-105 is known to be responsible for a number of processes including mediating RNA binding, interacting with nucleosomes, recruiting NEP (NS1), and involvement in viral assembly [3]. Of these 6 residues, residues 102, 104, and 105 are found in the conserved region. Also, 11 of the 32 residues (residues 47, 48, 69, 87, 91, 104, 105, 107, 108, 110, 141) are identified as part of the stacked interface of the oligomeric structure of M1 [4].

## Conserved regions in M2

The region found on the surface of M2 protein is made up of 9 residues (p-value = 0.05, connectivity 3.8). This region is comprised primarily of the transmembrane helix and includes residues 37, 38, 40, 41, 44, 45, and 46 [5]. From this set, residues 37 and 41 are known to work in tandem for proton selectivity [6]. There is also an intra-protein interaction between residues 41 and 44 (via indirect hydrogen-bonding), and between residues 44 and 45 (via a salt bridge) [7], resulting in the stabilization of the helix [8].

## Conserved regions in NA

There is a region of size 4 (p-value = 0.05, connectivity 1.5) on the NA protein. Using DOMMINO (PDB: 3B7E), we found that this small region is used in NA-NA binding and that every residue of the region is involved. The binding region is likely far larger than 4 residues, but this region represents a portion of this binding region.

## Conserved regions in NP

The two regions found on the surface of the NP protein are comprised of 50 residues (p-value = 0.00005, connectivity 3.0) and 18 residues (p-value = 0.03, connectivity 1.7). This first region has three main points of interest: residues 154, 155, 161, 162, and 164 that are responsible for RNA [9] and PB2 [10] binding; residues 201, 210, 211, 214, 215, 216, 217, and 219 that are responsible for NP-NP homo-oligomerization [11]; residues 250, 252, 253, 256, 260, 345, 440, 442, and 445 that are responsible for NP binding [11] to PB2 [10]. In addition to these functions, residue 373 is involved in recognition by the host T cell [12]. The second region had similar functions: residues 180, 181, 185, 227, 231, and 234 are involved in NP-NP homo-oligomerization [11].

## Conserved regions in NS1

The region found on the surface of the NS1 protein is comprised of 14 residues (p-value = 0.00005, connectivity 3.7) with residues 31, 35, 38, 41, 45, 46, and 49 specifically involved in dimerization and dsRNA interactions [13]. Residues 38 and 41 are involved directly with dsRNA binding via the backbone of the RNA molecule [13], residue 38 is required for binding, whereas residue 41 is not required, but greatly affects the binding affinity [13].

## Conserved regions in NS2

The region found on the surface of NS2 protein is comprised of 20 residues (p-value = 0.06, connectivity 3.8) and has a multifunctional domain. Residues 65, 66, 71, and 72 are involved in RNA binding and residues 65, 66, 71, 72, 74, 75, 76, 78, and 79 are involved in M1 binding [14] (residue 78 participates in a known interaction with M1) [15]. Interestingly, residues 65, 66, 71, and 72 are involved in both RNA and M1 binding. Moreover, the overlapping residues in the region all have side chains containing amino groups. Many of these amino groups are positively charged at cellular pH, while the others are available for hydrogen bonding. This is consistent with the fact that RNA has a negatively charged backbone. Immediately beyond the region overlap one can observe two negative charges that are involved in the electrostatic interaction between NS2 and the positively charged N-terminus of M1 [16]. This arrangement of charges may provide some insights into the dual functionality of this region.

Although this region is slightly over the desired p-value (α<0.05), it is clear by this overlap in functional regions that this region is biologically significant. This region also contains all conserved residues found on the surface of the protein model. Thus, given the model's small size, it is difficult to form two disconnected regions with sizes adding up to 20. This results in an irregular distribution, making it difficult to strictly constrain the p-value.

## Conserved regions in PA (1)

The region found on the surface of PA protein is comprised of 15 residues (p-value = 0.04, connectivity 3.1), which contains the last residues of the N-terminus. This portion of the N-terminus (residues 189, 192, and 193) is involved in PB1 binding [17]. This interaction is essential for the formation of the RNA Polymerase.

## Conserved regions in PA (2)

There were four significant regions on the surface of the PA: size 31 (p-value = 0.001, connectivity 3.4), size 22 (p-value = 0.008, connectivity 3.0), size 16 (p-value = 0.03, connectivity 3.4), and size 14 (p-value = 0.05, connectivity 3.1). Each of these regions is involved either directly, or indirectly, with PB1 binding [17]. Using DOMMINO, PDB structure 2ZNL gives structural evidence that specific residues of the regions of size 31 (591, 594, and 598) and size 22 (620, 623, 625, 628, 630, 663, 667, 670, 673, 674, 713, and 715) interact directly with PB1.

## Conserved regions in PB1 (1)

There were two regions of biological significance on PB1: one of size 7 (p-value = 0.08, connectivity 3.1) and one of size 6 (p-value = 0.1, connectivity 3.7). These two regions are on a very small structural fragment, which results in a very small available surface. We can deduce from the template structure (PDB: 3A1G) that these two regions are involved in interaction with PB2.

## Conserved regions in PB1 (2)

There is a region of size 11 (p-value = 0.3, connectivity 4.4) on the surface of PB1. This fragment of PB1 consists of only the last 15 N-terminal residues, 11 of which are conserved. Thus, each conserved residue is in this region; in fact, only 4 structurally mapped residues were not in the region. This means that there are only 1365 possible combinations of residues, yet we performed 10,000 iterations. Also, given this limited sampling size, it is highly probable that any 11 residues would result in a large region. Using DOMMINO (PDB: 2ZNL), we see that all 11 residues of this region are involved in PA binding.

## Conserved regions in PB2 (1)

There is a region on the surface of PB2 of size 24 (p-value = 0.001, connectivity 3.1). This region has a region that is associated with RNA cap-binding [18]. This is required for genome replication and expression.

## Conserved regions in PB2 (2)

There is a region on PB2 of size 16 (p-value = 16, connectivity 2.8). This region is entirely involved in cap-binding [19]. This has been structurally verified.

## Conserved regions in PB2 (3)

There were two regions on PB2: one of size 13 (p-value = 0.5, connectivity 5.1) and another of size 10 (p-value = 0.6, connectivity 5.2). Both of the regions are located on the C-terminal, which is involved in PB1 binding (determined from PDB ID 2ZTT using DOMMINO server). These regions have high p-values, but this is due to the fact that 23 of 29 surface residues are conserved. All of the conserved exterior residues are contained in these two regions. In fact, the highest possible p-value is 0.2.

# REFERENCES

1. Stevens J, Corper AL, Basler CF, Taubenberger JK, Palese P, et al. (2004) Structure of the uncleaved human H1 hemagglutinin from the extinct 1918 influenza virus. Science 303: 1866-1870.

2. Sha B, Luo M (1997) Structure of a bifunctional membrane-RNA binding protein, influenza virus matrix protein M1. Nat Struct Biol 4: 239-244.

3. Noton SL, Medcalf E, Fisher D, Mullin AE, Elton D, et al. (2007) Identification of the domains of the influenza A virus M1 matrix protein required for NP binding, oligomerization and incorporation into virions. J Gen Virol 88: 2280-2290.

4. Harris A, Forouhar F, Qiu S, Sha B, Luo M (2001) The crystal structure of the influenza matrix protein M1 at neutral pH: M1-M1 protein interfaces can rotate in the oligomeric structures of M1. Virology 289: 34-44.

5. Kochendoerfer GG, Salom D, Lear JD, Wilk-Orescan R, Kent SB, et al. (1999) Total chemical synthesis of the integral membrane protein influenza A virus M2: role of its C-terminal domain in tetramer assembly. Biochemistry 38: 11905-11913.

6. Phongphanphanee S, Rungrotmongkol T, Yoshida N, Hannongbua S, Hirata F (2010) Proton transport through the influenza A M2 channel: three-dimensional reference interaction site model study. J Am Chem Soc 132: 9782-9788.

7. Stouffer AL, Acharya R, Salom D, Levine AS, Di Costanzo L, et al. (2008) Structural basis for the function and inhibition of an influenza virus proton channel. Nature 451: 596-599.

8. Wang J, Qiu JX, Soto C, Degrado WF (2011) Structural and dynamic mechanisms for the function and inhibition of the M2 proton channel from influenza A virus. Curr Opin Struct Biol.

9. Kobayashi M, Toyoda T, Adyshev DM, Azuma Y, Ishihama A (1994) Molecular dissection of influenza virus nucleoprotein: deletion mapping of the RNA binding domain. J Virol 68: 8433-8436.

10. Biswas SK, Boutz PL, Nayak DP (1998) Influenza virus nucleoprotein interacts with influenza virus polymerase proteins. J Virol 72: 5493-5501.

11. Elton D, Medcalf E, Bishop K, Digard P (1999) Oligomerization of the influenza virus nucleoprotein: identification of positive and negative sequence elements. Virology 260: 190-200.

12. Valkenburg SA, Gras S, Guillonneau C, La Gruta NL, Thomas PG, et al. (2010) Protective efficacy of cross-reactive CD8+ T cells recognising mutant viral epitopes depends on peptide-MHC-I structural interactions and T cell activation threshold. PLoS Pathog 6: e1001039.

13. Wang W, Riedel K, Lynch P, Chien CY, Montelione GT, et al. (1999) RNA binding by the novel helical domain of the influenza virus NS1 protein requires its dimer structure and a small number of specific basic amino acids. RNA 5: 195-205.

14. Darapaneni V, Prabhaker VK, Kukol A (2009) Large-scale analysis of influenza A virus sequences reveals potential drug target sites of non-structural proteins. J Gen Virol 90: 2124-2133.

15. Akarsu H, Burmeister WP, Petosa C, Petit I, Muller CW, et al. (2003) Crystal structure of the M1 protein-binding domain of the influenza A virus nuclear export protein (NEP/NS2). EMBO J 22: 4646-4655.

16. Boulo S, Akarsu H, Ruigrok RW, Baudin F (2007) Nuclear traffic of influenza virus proteins and ribonucleoprotein complexes. Virus Res 124: 12-21.

17. Zurcher T, de la Luna S, Sanz-Ezquerro JJ, Nieto A, Ortin J (1996) Mutational analysis of the influenza virus A/Victoria/3/75 PA protein: studies of interaction with PB1 protein and identification of a dominant negative mutant. J Gen Virol 77 ( Pt 8): 1745-1749.

18. Honda A, Mizumoto K, Ishihama A (1999) Two separate sequences of PB2 subunit constitute the RNA cap-binding site of influenza virus RNA polymerase. Genes Cells 4: 475-485.

19. Guilligay D, Tarendeau F, Resa-Infante P, Coloma R, Crepin T, et al. (2008) The structural basis for cap binding by influenza virus polymerase subunit PB2. Nat Struct Mol Biol 15: 500-506.
