# Supplementary figures and images for: Extreme Evolutionary Conservation of Functionally Important Regions in H1N1 Influenza Proteome
Source: PLoS One. 2013 Nov 25;8(11):e81027. doi: 10.1371/journal.pone.0081027 (PMC3839886; doi:10.1371/journal.pone.0081027)

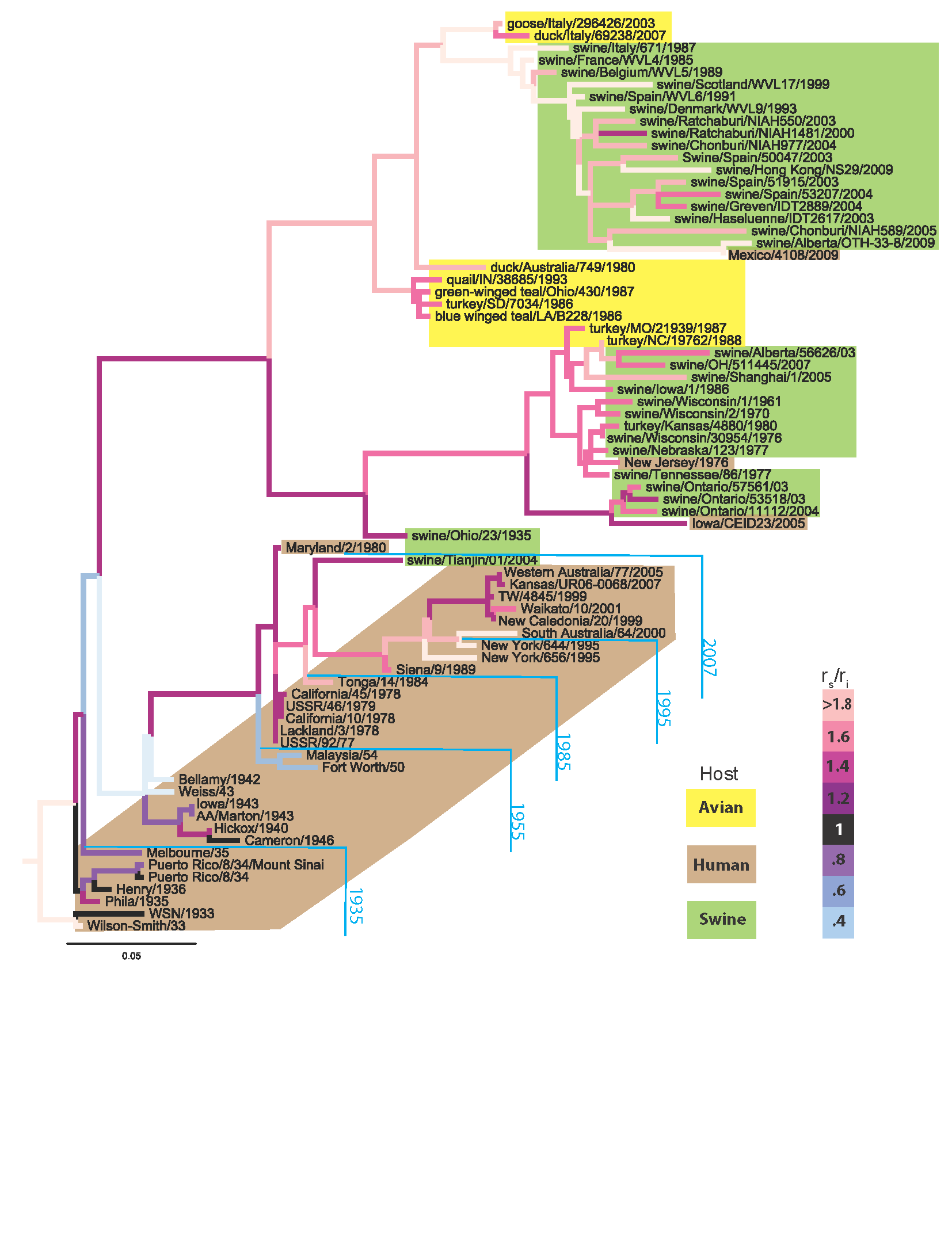

Supplement: Figure S1 — Phylogenetic relationships, species derivation and relative evolutionary rates inferred for the NA protein based on 75 accessions of H1N1 influenza. Shown are the inferred topology and the ratio of surface-to-interior amino acid substitutions (re/ri), calculated as the difference between the branch lengths estimated from the exterior and interior residues. The coloring scheme is the same as in Figure 1. (TIF) [file pone.0081027.s002.tif]

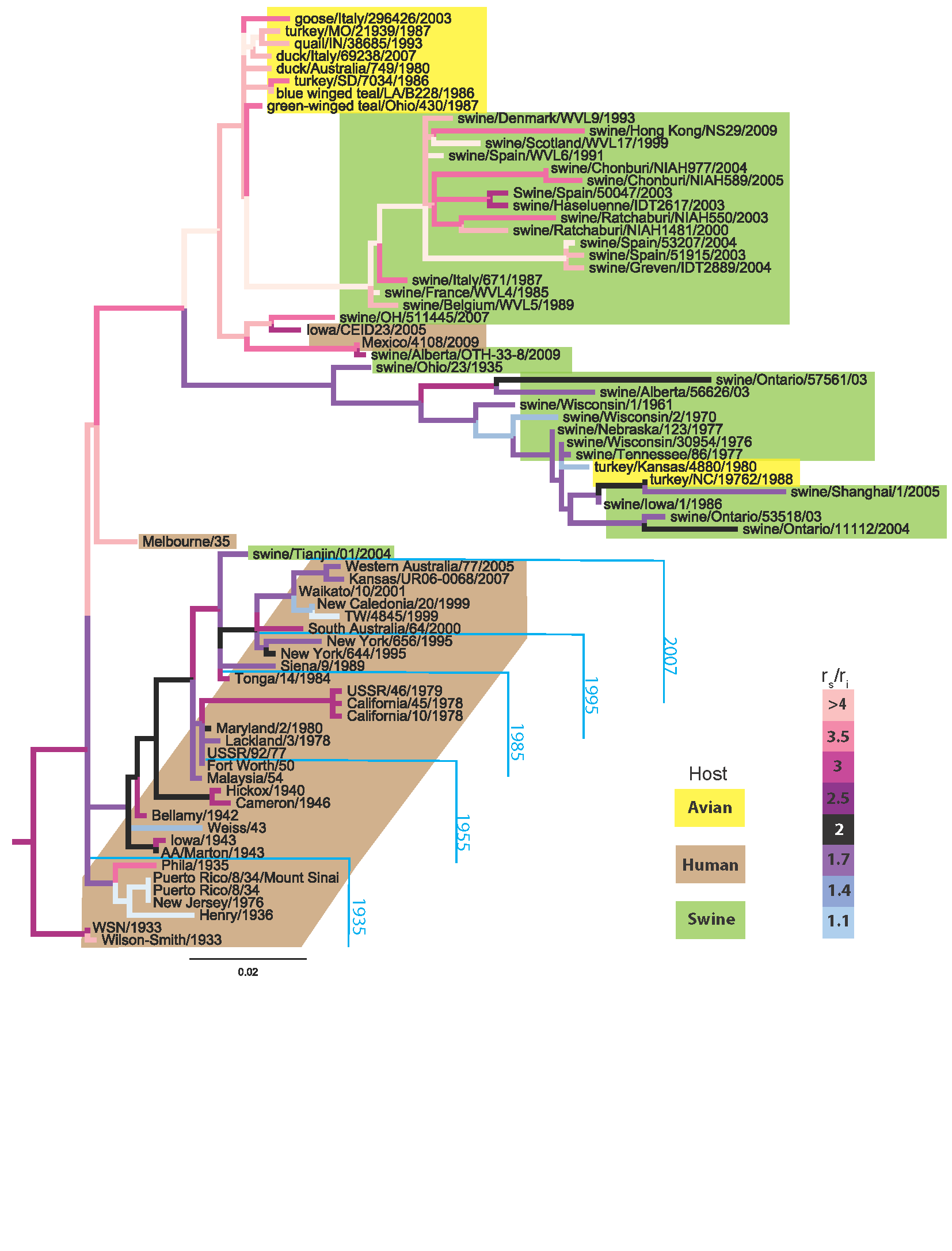

Supplement: Figure S2 — Phylogenetic relationships, species derivation and relative evolutionary rates inferred for the PA protein based on 75 accessions of H1N1 influenza. Shown are the inferred topology and the ratio of surface-to-interior amino acid substitutions (re/ri), calculated as the difference between the branch lengths estimated from the exterior and interior residues. The coloring scheme is the same as in Figure 1. (TIF) [file pone.0081027.s003.tif]

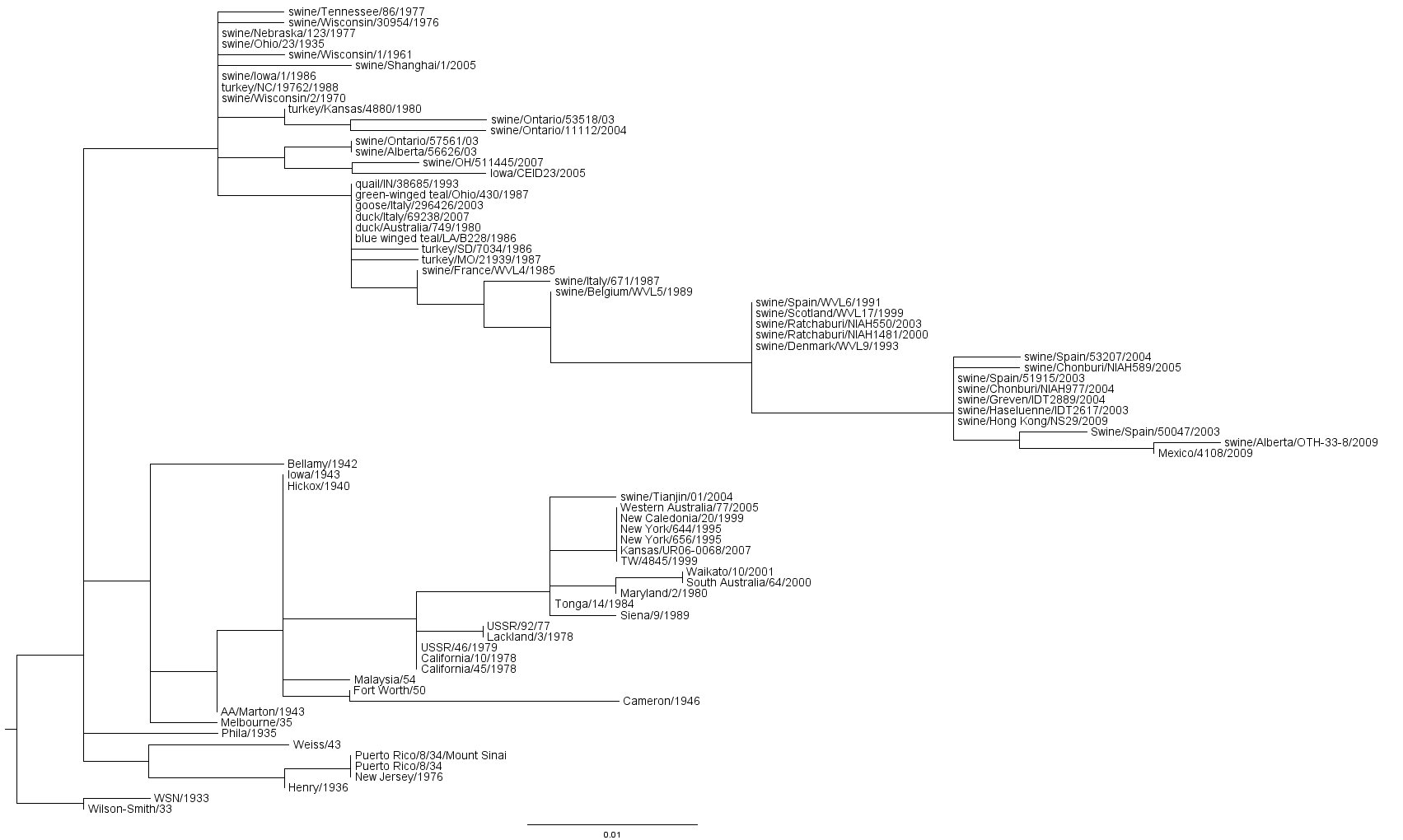

Supplement: Figure S3 — Phylogenetic relationships and relative evolutionary rates inferred for M1. The inferred topology and the ratio re/ri are calculated as in Figure 1. (TIF) [file pone.0081027.s004.tif]

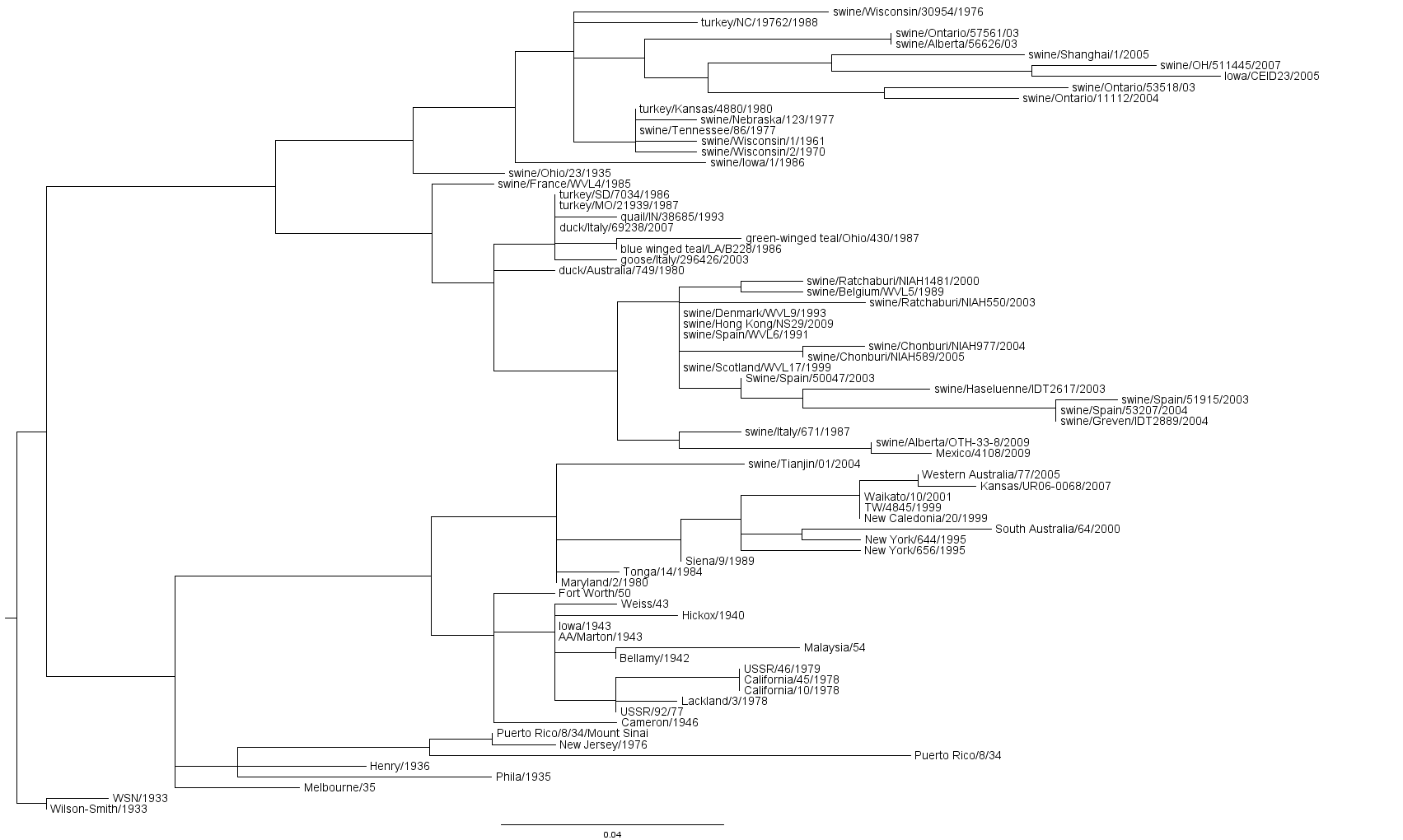

Supplement: Figure S4 — Phylogenetic relationships and relative evolutionary rates inferred for M2. The inferred topology and the ratio re/ri are calculated as in Figure 1. (TIF) [file pone.0081027.s005.tif]

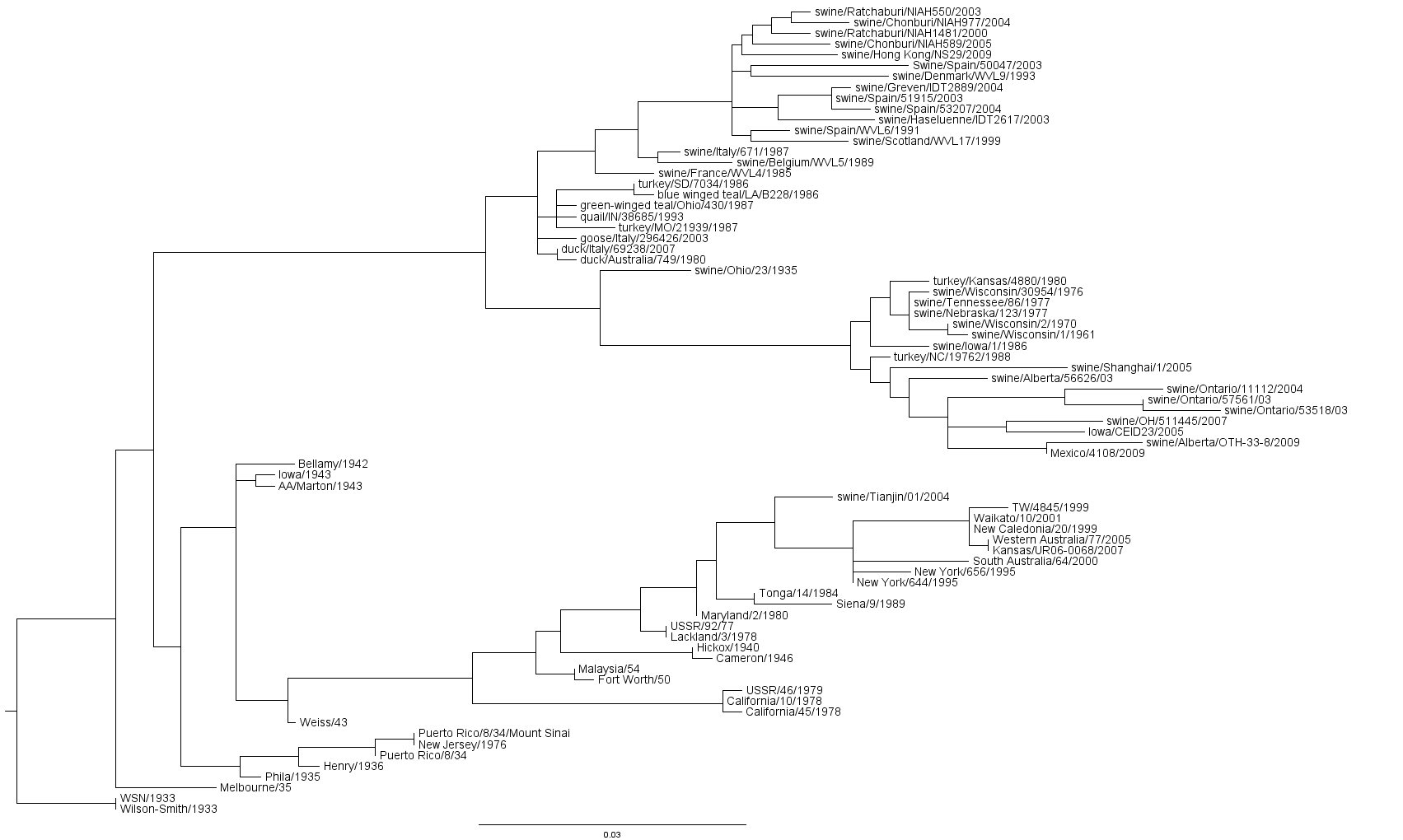

Supplement: Figure S5 — Phylogenetic relationships and relative evolutionary rates inferred for NP. The inferred topology and the ratio re/ri are calculated as in Figure 1. (TIF) [file pone.0081027.s006.tif]

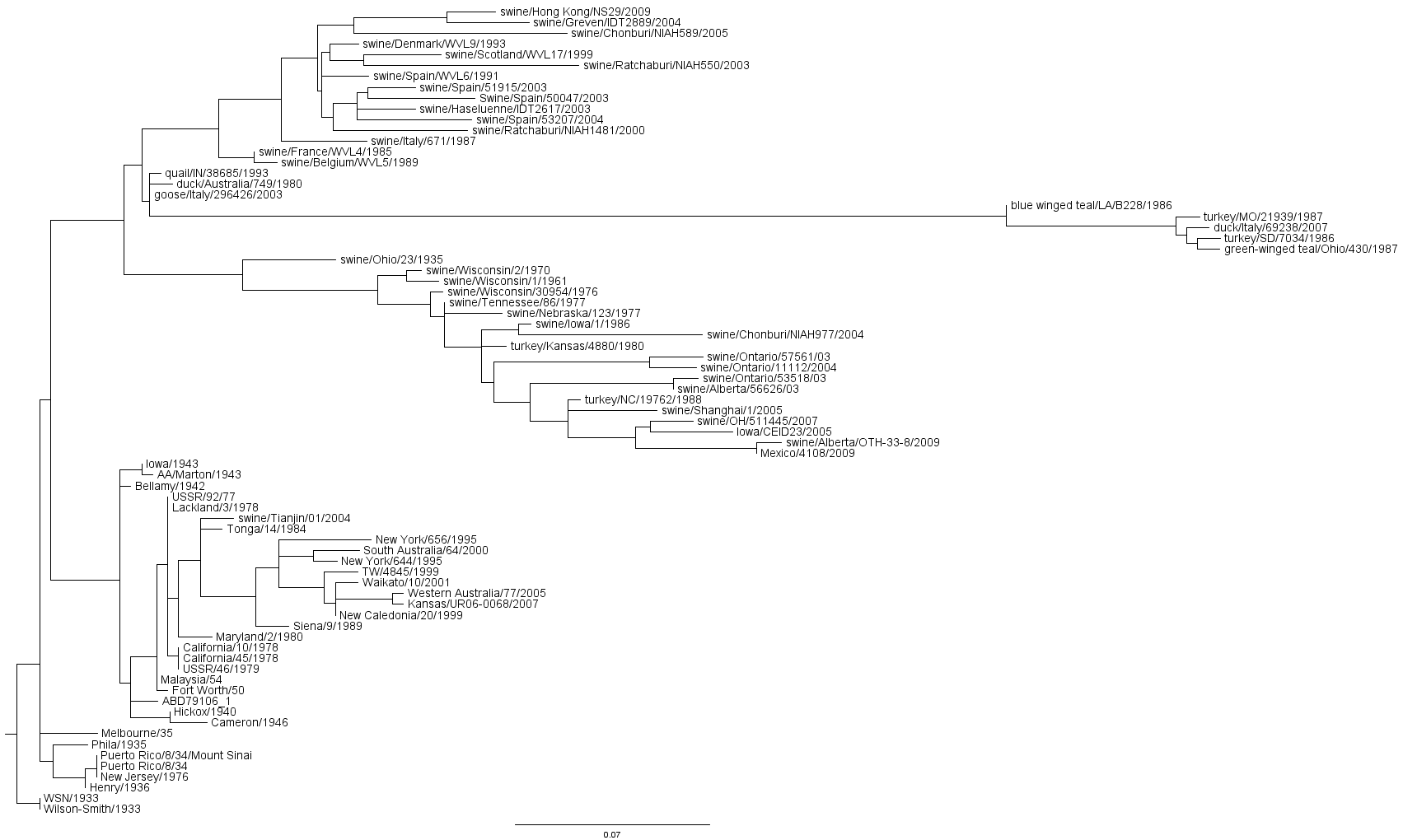

Supplement: Figure S6 — Phylogenetic relationships and relative evolutionary rates inferred for NS1. The inferred topology and the ratio re/ri are calculated as in Figure 1. (TIF) [file pone.0081027.s007.tif]

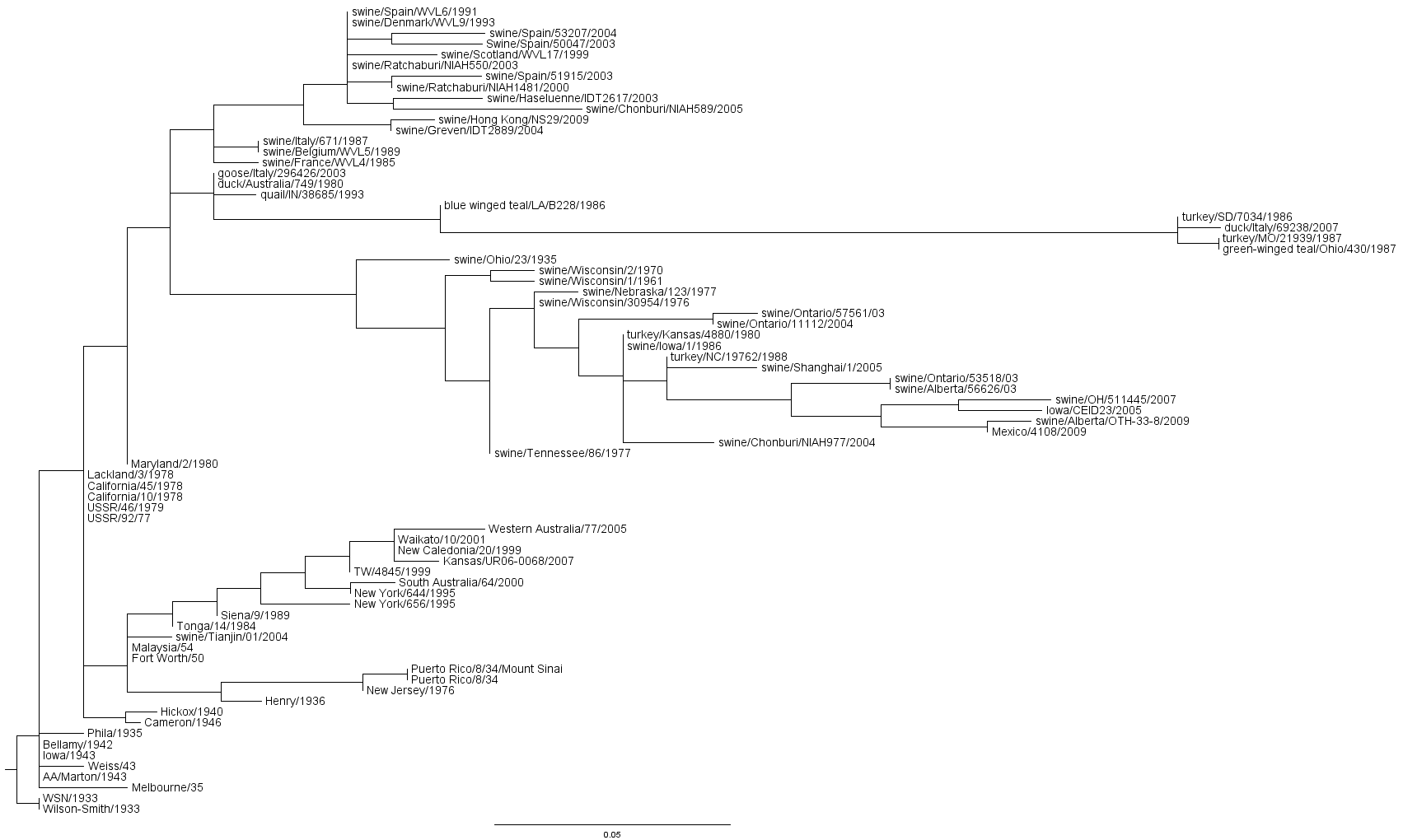

Supplement: Figure S7 — Phylogenetic relationships and relative evolutionary rates inferred for NS2. The inferred topology and the ratio re/ri are calculated as in Figure 1. (TIF) [file pone.0081027.s008.tif]

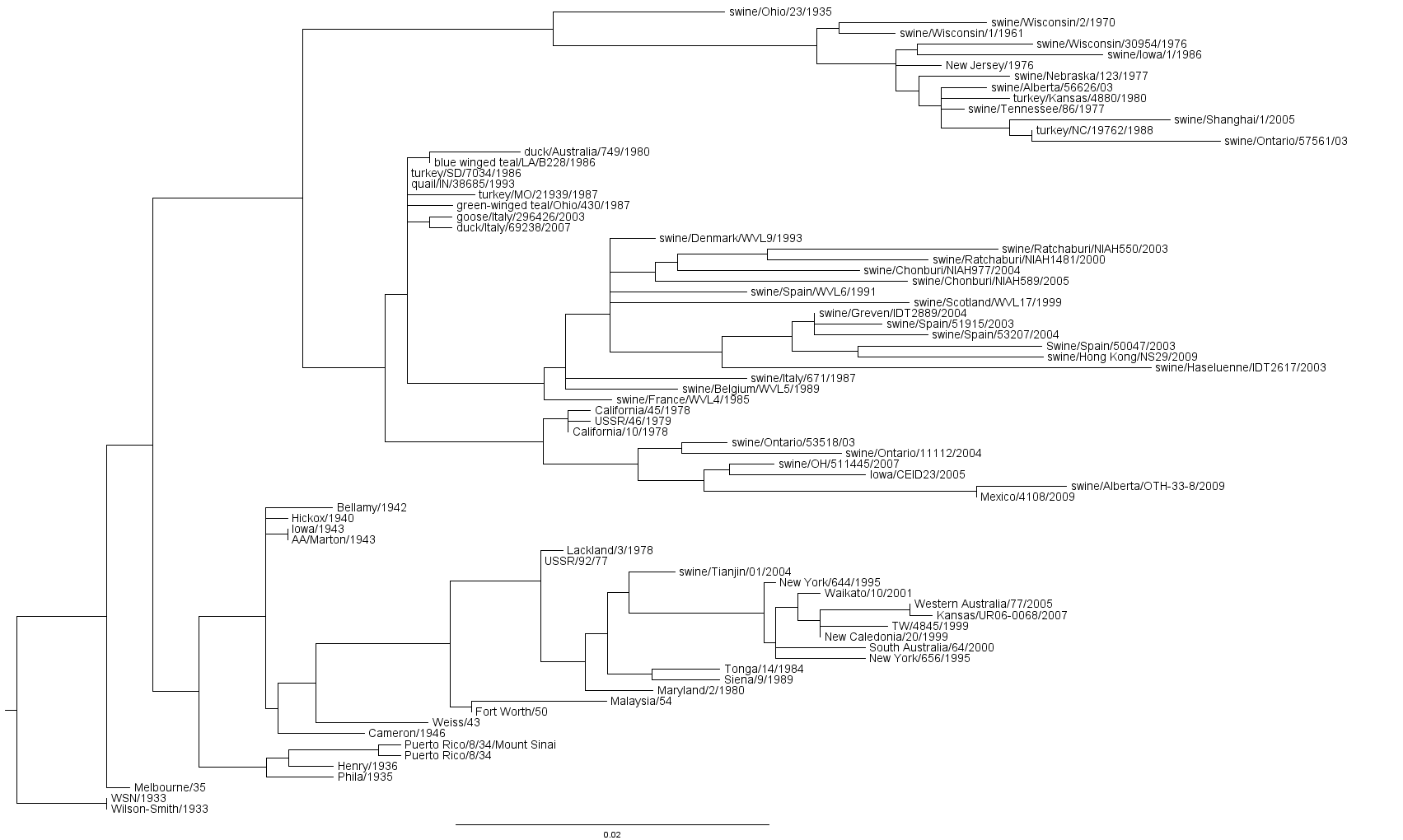

Supplement: Figure S8 — Phylogenetic relationships and relative evolutionary rates inferred for PB1. The inferred topology and the ratio re/ri are calculated as in Figure 1. (TIF) [file pone.0081027.s009.tif]

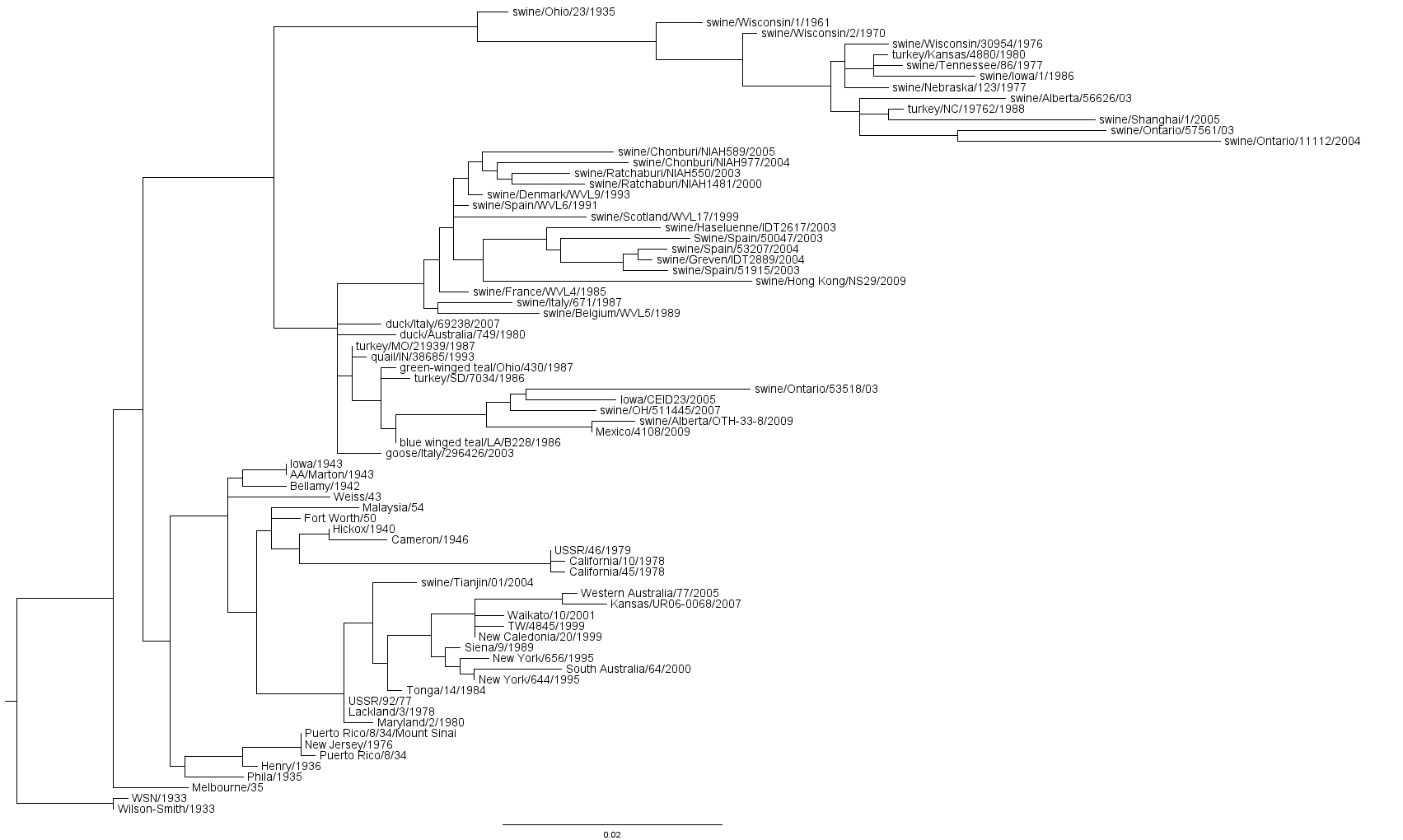

Supplement: Figure S9 — Phylogenetic relationships and relative evolutionary rates inferred for PB2. The inferred topology and the ratio re/ri are calculated as in Figure 1. (TIF) [file pone.0081027.s010.tif]

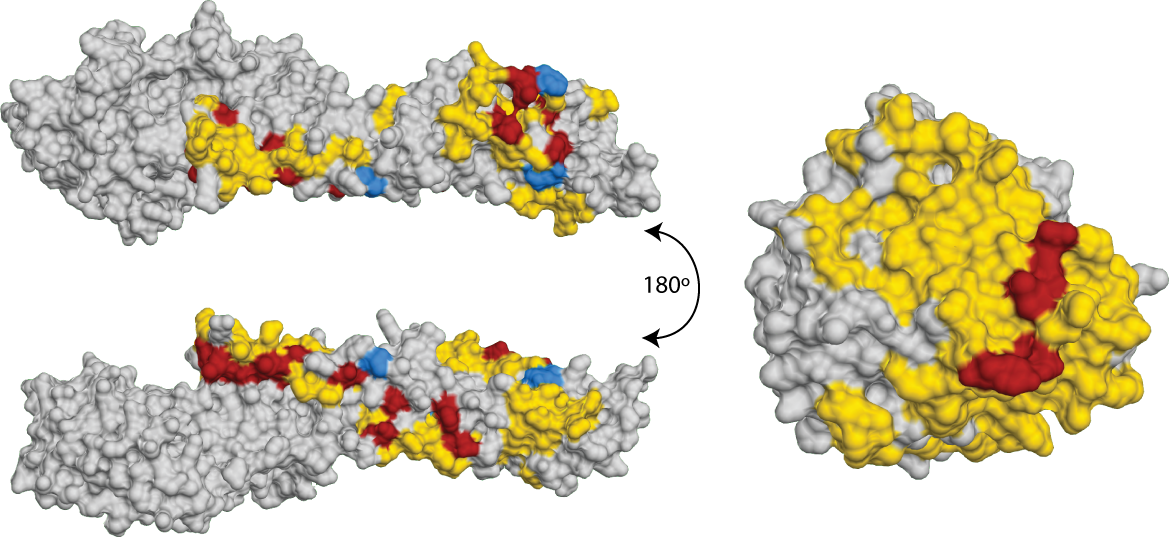

Supplement: Figure S10 — Overlap of binding sites and conserved regions of HA and NA proteins. Shown are conserved regions (blue), protein binding sites (gold) and the overlap of binding sites and conserved regions (red). (TIF) [file pone.0081027.s011.tif]
