# Supplementary material for: Extreme Evolutionary Conservation of Functionally Important Regions in H1N1 Influenza Proteome
Source: PLoS One. 2013 Nov 25;8(11):e81027. doi: 10.1371/journal.pone.0081027 (PMC3839886; doi:10.1371/journal.pone.0081027)
Supplement: Table S1 — A representative set of 75 strains used in our analysis. (DOCX) [file pone.0081027.s015.docx]

| Maryland/2/1980 | Phila/1935 | turkey/MO/21939/1987 |
| --- | --- | --- |
| swine/Tianjin/01/2004 | Puerto Rico/8/34/Mount Sinai | duck/Australia/749/1980 |
| Western Australia/77/2005 | Puerto Rico/8/34 | swine/Ohio/23/1935 |
| Kansas/UR06-0068/2007 | Henry/1936 | swine/Wisconsin/1/1961 |
| Waikato/10/2001 | Melbourne/35 | swine/Wisconsin/2/1970 |
| New Caledonia/20/1999 | Wilson-Smith/33 | New Jersey/1976 |
| TW/4845/1999 | WSN/1933 | turkey/Kansas/4880/1980 |
| South Australia/64/2000 | goose/Italy/296426/2003 | swine/Nebraska/123/1977 |
| New York/644/1995 | duck/Italy/69238/2007 | swine/Tennessee/86/1977 |
| New York/656/1995 | swine/Denmark/WVL9/1993 | swine/Wisconsin/30954/1976 |
| Siena/9/1989 | swine/Spain/53207/2004 | swine/Chonburi/NIAH977/2004 |
| Tonga/14/1984 | swine/Haseluenne/IDT2617/2003 | swine/Chonburi/NIAH589/2005 |
| USSR/46/1979 | Swine/Spain/50047/2003 | swine/Ontario/53518/03 |
| California/10/1978 | swine/Hong Kong/NS29/2009 | swine/Ontario/11112/2004 |
| California/45/1978 | swine/Spain/51915/2003 | swine/Ontario/57561/03 |
| Lackland/3/1978 | swine/Greven/IDT2889/2004 | swine/Alberta/56626/03 |
| USSR/92/77 | swine/Scotland/WVL17/1999 | swine/Iowa/1/1986 |
| Malaysia/54 | swine/Spain/WVL6/1991 | turkey/NC/19762/1988 |
| Fort Worth/50 | swine/France/WVL4/1985 | swine/Shanghai/1/2005 |
| Hickox/1940 | swine/Italy/671/1987 | swine/Ratchaburi/NIAH550/2003 |
| Cameron/1946 | swine/Belgium/WVL5/1989 | swine/Ratchaburi/NIAH1481/2000 |
| Iowa/1943 | green-winged teal/Ohio/430/1987 | Iowa/CEID23/2005 |
| AA/Marton/1943 | blue winged teal/LA/B228/1986 | swine/OH/511445/2007 |
| Bellamy/1942 | quail/IN/38685/1993 | swine/Alberta/OTH-33-8/2009 |
| Weiss/43 | turkey/SD/7034/1986 | Mexico/4108/2009 |
