# Supplementary material for: Extreme Evolutionary Conservation of Functionally Important Regions in H1N1 Influenza Proteome
Source: PLoS One. 2013 Nov 25;8(11):e81027. doi: 10.1371/journal.pone.0081027 (PMC3839886; doi:10.1371/journal.pone.0081027)
Supplement: Table S2 — Conserved regions with p-value and residues. Each protein is shown with their associated regions, p-values, and residues. Residues in bold are also found in intra-viral binding regions. (DOCX) [file pone.0081027.s016.docx]

| Protein | Region size | | p-value | Residues |
| --- | --- | --- | --- | --- |
| HA | | 9 | 0.005 | **344**,**345**,346,**347**,**349**,**354**,**355**,475,**476** |
|  | | 7 | 0.02 | **413**,**417**,**420**,**424**,**427**,**428**,**430** |
|  | | 7 | 0.02 | **386**,**397**,**438**,441,**446**,450,**453** |
| M1 | | 32 | 0.05 | 36,38,40,44,**47**,**48**,**69**,72,73,75,78,81,85,**87**,88,90,**91**,93,94,98,**102**,**104**,**105**,**107**,**108**,**110**,114,118,120,134,135,**141** |
| M2 | | 9 | 0.05 | **37**,**38**,**40**,**41**,**44**,**45**,**46**,49,51 |
| NA | | 4 | 0.05 | **107**,**108**,**113**,**170** |
| NP | | 50 | 0.00005 | **154**,**155**,158,**161**,**162**,**164**,168,171,**201**,204,205,209,**210**,**211**,**214**,**215**,**216**,**217**,  **219**,**250**,**252**,**253**,**256**,**260**,267,270,273,**345**,367,370,372,373,394,401,**440**,**442**,  **445**,466,467,480,482,484,487,490,493,497,498,499,500,502 |
|  | | 18 | 0.03 | 79,80,87,86,84,88,91,92,93,95,96,**180**,**181**,184,**185**,**227**,**231**,**234** |
| NS1 | | 14 | 0.00005 | 12,17,46,41,37,30,29,**31**,**35**,**38**,40,**45**,**49**,52 |
| NS2 | | 20 | 0.06 | **65**,**66**,**71**,**72**,**74**,**75**,**76**,**78**,**79**,90,91,92,94,95,97,101,105,108,112,114 |
| PA (1) | | 15 | 0.04 | 34,124,125,126,128,132,133,136,139,141,151,**189**,190,**192**,**193** |
| PA (2) | | 31 | 0.001 | 330,367,368,464,471,474,475,477,508,510,512,513,514,515,573,576,579,582,  583,586,587,590,**591**,**594**,597,**598**,601,603,604,605,606 |
|  | | 22 | 0.008 | **620**, **623**,**625**, **628**, **630**,656,659,660,**663**,664,**667**,**670**,**673**,  **674**,676,677,679,680,681,682,**713**,**715** |
|  | | 16 | 0.03 | 372,373,374,375,376,377,378,379,380,381,384,389,390,393,398,692 |
|  | | 14 | 0.05 | 242,243,245,247,248,249,250,252,255,257,259,260,264,266 |
| PB1 (1) | | 7 | 0.08 | 720,724,727,731,732,733,**734** |
|  | | 6 | 0.1 | **686**,688,**689**,**690**,**692**,**695** |
| PB1 (2) | | 11 | 0.3 | **1**,**2**,**3**,**4**,**5**,**7**,**8**,**9**,**10**,**13**,**15** |
| PB2 (1) | | 24 | 0.001 | **543**,**548**,**569**,**573**,**576**,578,579,582,634,639,641,642,643,644,646,652,654,660,  662,663,664,669,670,671 |
| PB2 (2) | | 16 | 0.02 | **343**,**345**,**349**,**353**,**408**,**412**,**415**,**417**,**419**,**421**,**423**,**425**,**426**,**427**,**429**,**448** |
| PB2 (3) | | 13 | 0.5 | 1,**3**,**4**,**5**,**8**,13,14,**15**,**17**,**18**,**19**,**20**,**21** |
|  | | 10 | 0.6 | **25**,**26**,**27**,28,30,**31**,33,**34**,**35**,36 |
