# Supplementary material for: Extreme Evolutionary Conservation of Functionally Important Regions in H1N1 Influenza Proteome
Source: PLoS One. 2013 Nov 25;8(11):e81027. doi: 10.1371/journal.pone.0081027 (PMC3839886; doi:10.1371/journal.pone.0081027)
Supplement: Table S3 — Outlying strains and the regions they affect. (DOCX) [file pone.0081027.s017.docx]

| Strain | Protein | Residue(s) |
| --- | --- | --- |
| A/duck/Italy/281904/2006 | NP | 219, 412 |
|  | NS1 | 41 |
|  | PB2 (2) | 353 |
| A/Managua/2860.01/2008 | NS2 | 76 |
|  | PA (2) | 330 |
|  | PB1 (1) | 717 |
| A/Managua/4412.01/2008 | HA | 29 |
|  | NS2 | 76 |
|  | PA (2) | 330 |
| A/swine/England/WVL15/1997 | NP | 168 |
|  | PA (2) | 713 |
|  | PB2 (2) | 353, 448 |
| A/swine/England/WVL16/1998 | NP | 168 |
|  | PA (2) | 551, 713 |
|  | PB2 (2) | 353 |
|  | PB2 (3) | 21 |
| A/swine/Iowa/1/1977 | NS1 | 167 |
|  | PA (2) | 559 |
|  | PB2 (1) | 634 |
| A/swine/Kyoto/3/1979 | PA (2) | 351, 379, 559 |
|  | PB2 (1) | 660 |
| A/swine/Minnesota/5892-7/1979 | NA | 272 |
|  | PA (2) | 559 |
|  | PB2( 1) | 660 |
| A/swine/Tennessee/37/1977 | M1 | 33 |
|  | PA (2) | 559 |
|  | PB2 (1) | 660 |
| A/swine/Wisconsin/1915/1988 | HA | 376 |
|  | NP | 116, 216 |
|  | PA (2) | 559, 603 |
| A/Texas/UR06-0217/2007 | NS2 | 76 |
|  | PA (1) | 133 |
|  | PB2 (1) | 664 |
|  | PB2 (3) | 14 |
| A/TW/130/1996 | M1 | 5 |
|  | NA | 113 |
|  | NP | 500 |
| A/TW/3355/1997 | NP | 292, 298, 500 |
|  | PA (1) | 7 |
| A/Zhejiang/2/2009 | HA | 428 |
|  | M1 | 98 |
|  | PA (2) | 419 |
